# Linking indirect effects of cytomegalovirus in transplantation to modulation of monocyte innate immune function

**DOI:** 10.1126/sciadv.aax9856

**Published:** 2020-04-22

**Authors:** Pritha Sen, Adrian R. Wilkie, Fei Ji, Yiming Yang, Ian J. Taylor, Miguel Velazquez-Palafox, Emilia A. H. Vanni, Jean M. Pesola, Rosio Fernandez, Han Chen, Liza M. Morsett, Erik R. Abels, Mary Piper, Rebekah J. Lane, Suzanne E. Hickman, Terry K. Means, Eric S. Rosenberg, Ruslan I. Sadreyev, Bo Li, Donald M. Coen, Jay A. Fishman, Joseph El Khoury

**Affiliations:** 1 Center for Immunology and Inflammatory Diseases, Massachusetts General Hospital and Harvard Medical School, Boston, MA, USA.; 2 Transplant Infectious Disease and Compromised Host Program, Division of Infectious Diseases, Massachusetts General Hospital and Harvard Medical School, Boston, MA, USA.; 3 Department of Biological Chemistry and Molecular Pharmacology, Blavatnik Institute, Harvard Medical School, Boston, MA, USA.; 4 Department of Molecular Biology and Department of Pathology, Massachusetts General Hospital and Harvard Medical School, Boston, MA, USA.; 5 Klarman Cell Observatory, Broad Institute of MIT and Harvard, Cambridge, MA, USA.; 6 BD Life Sciences—Informatics, Ashland, OR, USA.; 7 Department of Neurology and Center for Molecular Imaging Research, Department of Radiology, Massachusetts General Hospital and Program in Neuroscience, Harvard Medical School, Boston, MA, USA.; 8 Harvard Bioinformatics Core, Harvard TH Chan School of Public Health, Boston, MA, USA.; 9 Autoimmunity Cluster, Immunology and Inflammation Research Therapeutic Area, Sanofi, Cambridge, MA, USA.

## Abstract

Cytomegalovirus (CMV) is an important cause of morbidity and mortality in the immunocompromised host. In transplant recipients, a variety of clinically important “indirect effects” are attributed to immune modulation by CMV, including increased mortality from fungal disease, allograft dysfunction and rejection in solid organ transplantation, and graft-versus-host-disease in stem cell transplantation. Monocytes, key cellular targets of CMV, are permissive to primary, latent and reactivated CMV infection. Here, pairing unbiased bulk and single cell transcriptomics with functional analyses we demonstrate that human monocytes infected with CMV do not effectively phagocytose fungal pathogens, a functional deficit which occurs with decreased expression of fungal recognition receptors. Simultaneously, CMV-infected monocytes upregulate antiviral, pro-inflammatory chemokine, and inflammasome responses associated with allograft rejection and graft-versus-host disease. Our study demonstrates that CMV modulates both immunosuppressive and immunostimulatory monocyte phenotypes, explaining in part, its paradoxical “indirect effects” in transplantation. These data could provide innate immune targets for the stratification and treatment of CMV disease.

## Teaser

CMV-infected monocytes are unable to phagocytose fungal pathogens yet produce inflammation associated with rejection and GVHD.

## INTRODUCTION

Human cytomegalovirus (CMV) is a ubiquitous b-herpes virus that causes substantial morbidity and mortality in the immunocompromised host (*1*). In solid organ and bone marrow transplant recipients, in addition to causing multi-organ tissue invasive disease, CMV has been associated with “indirect effects,” a term encapsulating a diverse group of clinically important, paradoxically immunosuppressive and immunostimulatory phenotypes attributed to immune modulation by viral infection. These effects are most pronounced in the setting of primary infection and serological discordance between CMV donor (D^+^) and recipient (R^−^) immunoglobulin G (IgG) (*2*). These indirect effects include the immunosuppressive phenotype of increased predisposition to additional opportunistic infections and mortality from invasive fungal disease (IFD) (*3*, *4*). CMV is also associated with allograft rejection and organ-specific allograft injury in solid organ transplantation (SOT), including accelerated coronary vasculopathy in cardiac transplantation, bronchiolitis obliterans syndrome in lung transplantation, vanishing bile duct syndrome in liver transplantation, and graft-versus-host disease (GVHD) in bone marrow transplantation (BMT), all sequelae thought to result from a CMV-mediated immunostimulatory milieu (*2*, *4*–*8*). While these indirect effects of CMV have been generally attributed to virally mediated immune modulation, the mechanisms underlying the immunosuppressive increased risk of IFD and the immunostimulatory phenotype of allograft rejection and dysfunction are not well understood.

Monocytes, innate immune cells essential for host defense against pathogens, have a critical role in CMV pathogenesis. Monocytes are one of the primary cellular targets for CMV, are highly permissive to acute infection with CMV, facilitate hematogenous dissemination of virus into tissues, and are a reservoir of latent virus (*9*–*11*). There has been substantial investigation into understanding the monocyte-CMV host-pathogen interface from the host perspective; however, these previous studies have focused primarily on how CMV alters monocyte chemotaxis and migration, differentiation and polarization into macrophages, cytokine and chemokine production, and evasion of apoptosis and other cellular fate pathways (*12*–*15*).

Given that monocytes are also responsible for mediating antifungal immunity (*16*), we hypothesized that CMV-mediated dysregulation of monocyte transcriptional networks and effector functions could lead to increased vulnerability to secondary infections, as observed in transplant recipients infected by CMV, and also could be a potential link between the innate immune system and allograft rejection. In this study, we performed unbiased bulk and single-cell transcriptional profiling of CMV-infected monocytes in vitro and from heart transplant recipients and pair these data with functional analyses, identifying several pathways through which CMV modulates innate immune responses that have direct relevance to understanding indirect effects in transplantation.

## RESULTS

### Dissecting the monocyte-CMV host-pathogen interface using RNA sequencing

**Fig. 1:**
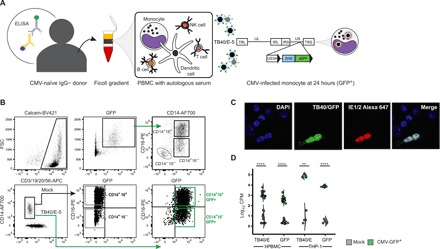
GFP expression is a marker of CMV-infected monocytes in an in vitro PBMC model of CMV infection. (**A**) PBMCs from CMV IgG-negative immunocompetent human donors were cultured with autologous serum and infected with TB40/E-5 for 24 hours as described. Schematic representation of the TB40/E-5 genome shows locations of unique long (UL) and unique short (US) regions flanked by terminal (TRL/TRS) and internal (IRL/IRS) inverted repeats and location of GFP expression cassette, driven by a SV40 promoter, between US34 and TRS1 genes. (**B**) Twenty-four hours after infection, GFP^+^ cells localized to the monocyte locations on standard forward/side scatter, and staining showed that most of the GFP^+^ cells belonged to CD14^+^ PBMCs. A small number of CD14^−^/16^−^/HLA-DR^+^/GFP^+^ CMV-infected circulating dendritic cells were also detected (fig. S2). (**C**) Calcein^+^/CD3^−^/19^−^/20^−^/56^−^/CD14^+^/16^−^ and CD14^+^/16^+^ cells from mock-infected PBMCs and CD14^+^/16^−^/GFP^+^ and CD14^+^/16^+^/GFP^+^ from CMV-infected PBMCs were sorted by FACS and used for all downstream RNA-Seq analysis. Co-staining by immunohistochemistry demonstrates that GFP colocalizes with CMV IE1/2 in PMA-stimulated THP-1 infected with TB40/E-5 24 hours after infection (Zeiss 63X). (**D**) Sequences aligning to the TB40/E-5 reference genome, graphed as counts per million (CPM), were present in CMV-infected primary PBMCs and THP-1 and either absent or low in mock-infected specimens (*P* < 0.0001 and *P* < 0.0001, respectively, unpaired *t* test). Sequences aligning to GFP were present in CMV-infected PBMCs and THP-1 cell line and either absent or low in mock-infected specimens (*P* = 0.0013 and *P* < 0.0001, respectively, unpaired *t* test). **P < 0.01 and ****P < 0.0001. PBMC, peripheral blood mononuclear cells; NK, natural killer; FSC, forward scatter; BV421, Brilliant Violet 421; AF700, AlexaFluor 700; APC, allophycocyanin; PE, phycoerythrin; GFP, green fluorescent protein; CPM, counts per million.

Peripheral blood mononuclear cells (PBMCs) isolated from immunocompetent CMV-seronegative donors were cultured in donor-autologous serum and then infected with TB40/E-5, a CMV strain with extended cellular tropism that produces green fluorescent protein (GFP) under the control of an SV40 promoter upon entry of the viral genome into the host cell nucleus (Fig. 1A) (*17*, *18*). After 24 hours of infection, we found GFP expression to be a marker for CMV infection of monocytes both by flow cytometry and by immunohistochemistry. Ninety percent of GFP^+^ cells co-stained with the monocyte marker CD14 (Fig. 1B and fig. S1) and GFP colocalized to cells expressing CMV immediate early proteins 1 and 2 (IE1/2) (Fig. 1C). CD14^+^/16^−^ and CD14^+^/16^+^ mock and CD14^+^/16^−^/GFP^+^ and CD14^+^/16^+^/GFP^+^ CMV-infected monocytes were sorted using flow-assisted cell sorting (FACS), and RNA sequencing (RNA-Seq) was performed on these enriched cell populations (Fig. 1B). Replicate samples from mock- and CMV-infected monocytes clustered distinctly both by the correlation of gene expression values and by principal components analysis (PCA) (fig. S2), attesting to the reproducibility of our approach. In this experimental design evaluating CMV-infected monocytes 24 hours after infection in vitro, RNA sequences aligning to the TB40/E-5 reference genome and GFP sequence were present in CMV-infected specimens and were either absent or low in mock specimens (Fig. 1D), and two-thirds of CMV viral transcripts detected in GFP^+^ monocytes belonged to the immediate-early and delayed-early family of CMV transcripts (fig. S3).

**Fig. 2:**
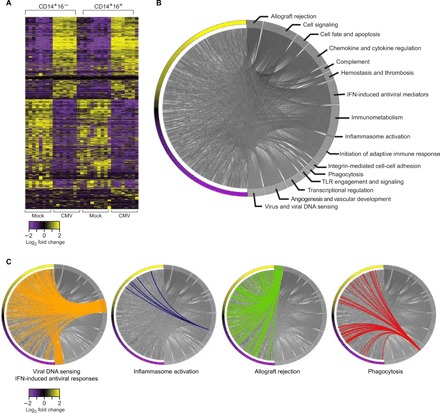
Unbiased sequencing defines a distinct monocyte-CMV transcriptional signature. (**A**) Heat map with hierarchical clustering showing genes differentially expressed in mock-infected CD14^+^/16^−^, CMV-infected CD14^+^/16^+^/GFP^+^, mock-infected CD14^+^/16^−^, and CMV-infected CD14^+^/16^−^/GFP^+^ monocytes. (**B**) Chord diagram illustrating GSEA of the 2167 differentially expressed transcripts in CMV-infected CD14^+^/16^−^ monocytes defines 16 categories of biological processes dysregulated in CMV-infected monocytes. (**C**) Chord diagrams highlighting differentially expressed genes within the MSigDB-Hallmarks and InnateDB summary terms viral DNA sensing and IFN-induced viral mediators, inflammasome activation, allograft rejection, and phagocytosis.

We performed differential gene expression analysis to compare the transcriptomes of CMV- and mock-infected monocytes from 10 donors and found 2167 and 2433 genes differentially expressed in the CD14^+^/16^−^ and CD14^+^/16^+^ monocyte subsets, respectively, at an adjusted false discovery rate (FDR) of <0.05. Hierarchical clustering of these differentially expressed genes showed a distinct transcriptional signature (Fig. 2A). We focused our subsequent analyses on those genes differentially expressed between mock- and CMV-infected CD14^+^/16^−^ monocytes. These historically categorized “classical” monocytes, defined by their role in mediating defense and inflammatory responses and their rapid recruitment to sites of injury and inflammation through chemotaxis, are the predominant circulating monocyte in humans. To analyze pathways involved in innate immunity, we performed gene set enrichment analysis (GSEA) by applying the GSEA tool against the MSigDB (*19*) and InnateDB (*20*) pathway databases. This analysis revealed 154 unique significantly enriched pathways (tables S1 and S2). We classified these 154 pathways into 16 subcategories, identifying the following biological processes affected by transcriptional changes in CMV-infected monocytes: allograft rejection, cellular signaling, cellular fate and death, chemokine and cytokine regulation, complement, hemostasis and thrombosis, interferon (IFN)–induced antiviral responses, immunometabolism, inflammasome activation, integrin-mediated cell-cell adhesion, phagocytosis, Toll-like receptor (TLR) engagement and signaling, transcriptional regulation, angiogenesis and vascular development, and virus and viral DNA sensing (Fig. 2B and table S3). The proteins governing these diverse group of biological processes whose transcripts were altered by CMV infection localize throughout the cell, spanning the extracellular space, cellular membrane, cytoskeleton, cytosol, mitochondria, Golgi–endoplasmic reticulum complex, phagosome-lysosome complexes, and perinuclear and nuclear cellular spaces, further emphasizing the impact of early CMV infection on cellular processes in these innate immune cells (fig. S4).

To understand how CMV-infected monocytes could mediate the indirect effects of increased morbidity and mortality from IFD and allograft rejection in transplantation, we focused on the expression profiles of genes within the GSEA-enriched pathways of viral DNA sensing and IFN-induced antiviral responses, inflammasome activation, allograft rejection, and phagocytosis (Fig. 2A). As expected, we found that pathways involved in viral DNA sensing and IFN-induced antiviral responses were biological processes enriched among up-regulated genes according to GSEA (Hallmark, Reactome, FDR = 0). Inflammasome activation was also enriched among up-regulated genes (Reactome, *P* = 6.7 × 10^−3^), while transcripts involved in allograft rejection also had a positive normalized enrichment score (Hallmark, FDR = 0.02). In contrast, several genes involved in phagocytosis were enriched among down-regulated genes [Kyoto Encyclopedia of Genes and Genomes (KEGG), *P* = 8.2 × 10^−4^]. These results suggest that overall, at a transcriptional level, the biological processes of viral DNA sensing and antiviral responses, inflammasome activation, and allograft rejection were induced, while biological processes associated with phagocytosis were inhibited in CMV-infected monocytes (Fig. 2C).

### CMV induces the expression of intracellular viral pattern recognition receptors and inhibits the expression of cell surface scavenger receptors

**Fig. 3:**
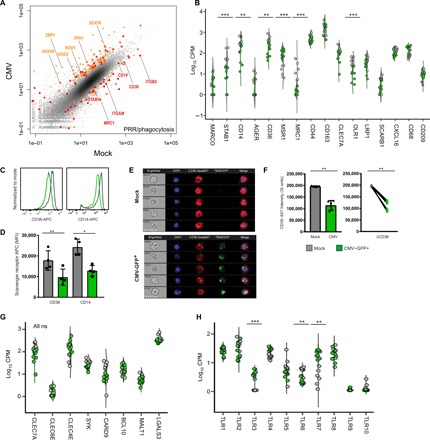
CMV infection of monocytes can concurrently induce the expression of cytoplasmic dsDNA viral PRRs and inhibit the surface expression of bacterial and fungal pathogen PRRs. (**A**) Scatter plot showing all transcripts detected by RNA-Seq in mock- and CMV-infected CD14^+^/16^−^ monocytes. Differentially expressed genes within curated lists related to viral PRRs (orange), nonviral PRRs (red), and phagocytosis (red) are highlighted. (**B**) Binary violin plots showing the mRNA expression of all scavenger receptors as determined by RNA-Seq and (**C** and **D**) protein expression of scavenger receptors CD14 and CD36 using conventional flow cytometry. Overlay histogram plots that demonstrate the MFI of allophycocyanin (APC), the marker for CD14 or CD36 expression in mock- and CMV-infected monocytes from one representative experiment, is shown. Whisker plots include all composite data from biological replicates (*n* = 4, geometric MFI ± SEM, CD14 *P* < 0.0024, CD36 *P* < 0.001, paired *t* test). (**E** and **F**) Protein expression of surface CD36 was confirmed using ImageStream (Amnis) with panels displaying five representative mock- and CMV-infected monocytes. Magnification, ×40. Scale bars, 10 μm. Whisker plots include composite data from all biological replicates (*n* = 5 cells, ImageStream units; *P* < 0.0001, unpaired *t* test). Binary violin plots showing the mRNA expression of (**G**) select C-type lectin (CLEC) receptors and (**H**) TLRs in mock- and CMV-infected CD14^+^/16^−^ monocytes. *P* values (C, D, and F) or FDR-adjusted *P* values (B, G, and H) are denoted as *P < 0.05, **P < 0.01, and ***P < 0.001. PRR, pattern recognition receptor; ns, not significant.

To delve further into the specific pathways through which CMV inhibits the biological process of phagocytosis, we manually curated a list of genes involved in bacterial, fungal, mycobacterial, and parasitic pathogen-associated molecular pattern (PAMP) recognition and marked these genes on a scatter plot together with genes involved in viral DNA sensing and phagocytosis pathways identified from MSigDB and InnateDB (Fig. 3A). Analysis of subsets of genes involved in these pathways revealed that intracellular double-stranded DNA (dsDNA) viral pattern recognition receptors (PRRs) were markedly induced in CMV-infected monocytes (Fig. 3A). This included increased expression of all transcripts encoding for DExD/ H-box RNA helicases of the RIG-like receptor (RLR) family, including *DDX58*/RIG-I [logFC (fold change) = 3.8, FDR = 1.8 × 10^−10^], *IFIH1*/MDA5 (logFC = 3.7, FDR = 9.2 × 10^−10^), and *DHX58*/LGP2 (logFC = 3.7, FDR = 6.8 × 10^−10^) (Fig. 3A) (*21*–*23*). *ZBP1*, the gene encoding the DNA-dependent activator of IFN-regulatory factors (DAI), a cytosolic viral DNA sensor, was also up-regulated (logFC = 6.4, FDR = 1.9 × 10^−20^). Expression of genes for nonobese diabetic mice (NOD)–like receptors (NLRs) *NOD1* and *NOD2*, which detect intracellular viral PAMPs, was also up-regulated in CMV-infected monocytes (logFC > 2.9, FDR < 2.5 × 10^−6^). CMV infection did not markedly alter the expression of tetraspanins, a family of surface transmembrane molecules implicated in viral pathogenesis.

In contrast to the increased expression of intracellular viral PRRs, there was marked down-regulation of several surface membrane PAMP receptors. The expression of complement receptor 3 (*CR3*, *ITGAM/ITGB2*), a receptor for CpG motifs and multiple fungal pathogens, including *Candida albicans*, *Cryptococcus neoformans*, *Aspergillus fumigatus*, and dimorphic fungi (*24*–*26*), was decreased (logFC = −3.3, FDR = 1.8 × 10^−7^) in CMV-infected monocytes. Expression of genes for several members of the scavenger receptor family known to recognize bacterial and fungal PAMPs was also significantly down-regulated in CMV-infected monocytes. These genes included *CD36*, which promotes phagocytosis of *C. albicans* and *C. neoformans* (*27*, *28*) (logFC = −1.8, FDR = 0.004), *MRC1*, which is shown to recognize both *C. albicans* and *Pneumocystis jirovecii* (*29*) (log −4.4, FDR = 5.4 × 10^−11^), and the lipopolysaccharide, lipoteichoic acid, and lipoarabinomannan receptor *CD14* (logFC = −1.7, FDR = 0.01) (Fig. 3, A and B) (*30*). Flow cytometry and ImageStream (Amnis) confirmed that the reduced expression of *CD14* and *CD36* mRNA levels correlated with reduced surface expression at the protein level, validating our RNA-Seq results (Fig. 3, C to F). Transcripts for *MSR1*, a scavenger receptor known to bind low-density lipoprotein (LDL) particles and β-amyloid fibrils, were up-regulated in CMV-infected monocytes (logFC = 2.4, FDR = 9.2 × 10^−5^), while expression of *OLR1*, the gene encoding for Lox-1, a scavenger receptor responsible for recognition of oxidized LDL, was significantly down-regulated (logFC = −2.1, FDR = 0.002), suggesting that CMV-mediated changes in scavenger receptor expression in monocytes does not uniformly change expression patterns of all genes within the same family (Fig. 3B).

Expression of *CLEC7A/*Dectin-1, *CLEC6A/*Dectin-2, and *CLEC4E/*Mincle, genes encoding C-type lectin (CLEC) receptors known to have a central role in mediating antifungal immunity, and *SYK*, *CARD9*, *BCL10*, *MALT1*, and *LGALS3*, intracellular molecules downstream to these canonical fungal PRRs, was not significantly changed in CMV-infected CD14^+^/16^−^ monocytes (FDR ≥ 0.14) (Fig. 3G). While other *CLEC* receptor genes *CD207*, *CLEC1B*, *CLEC3B*, *CLEC4A*, *CLEC5A*, *CLEC10A*, and *CLEC11A* were significantly down-regulated (logFC ≤ −2, FDR ≥ 0.002), these transmembrane receptors have not been directly implicated in regulating microbial pathogen recognition (*31*). Genes for TLR, which partner with both scavenger receptors and CLEC receptors to promote pathogen recognition and phagocytosis, showed a mixed pattern of dysregulation. In CMV-infected monocytes, there was simultaneous induction of expression of viral PRR genes *TLR3* (logFC = 3.9, FDR = 7.4 × 10^−9^) and *TLR7* (logFC = 1.8, FDR = 0.003) (*32*–*34*) and inhibition of expression of *TLR6* (logFC = −1.78, FDR = 0.01) (Fig. 3H), the latter of which has been shown to partner with TLR2 in the recognition of *C. albicans* and prevention of disseminated candidiasis and confer risk to developing invasive aspergillosis (*35*, *36*). CMV infection did not markedly alter the expression of genes involved in Fcγ receptor–mediated phagocytosis. Together, these data indicate that CMV infection of CD14^+^/16^−^ monocytes simultaneously induces the expression of multiple intracellular viral PRRs from the RLR, DAI, and NOD families while selectively down-regulating expression of surface scavenger receptors *CD36* and *MRC1*, complement receptor 3, and *TLR6*, all of which have a demonstrated role in fungal pathogen recognition and clearance (table S4).

### CMV-infected monocytes are unable to effectively phagocytose fungal pathogens

**Fig. 4:**
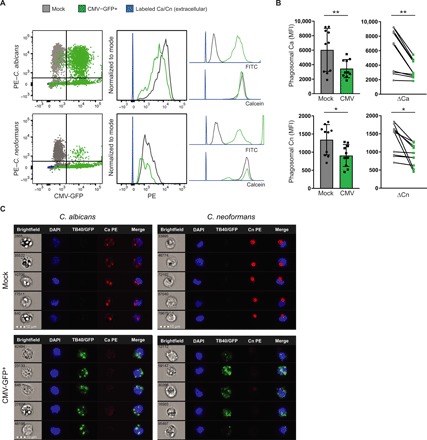
CMV-infected monocytes have a decreased phagocytic index for *C. albicans* and *C. neoformans*. pHrodo-red–labeled *C. albicans* (Ca) and *C. neoformans* (Cn) were incubated with mock- and CMV-infected PBMC cultures at a ratio of 5:1 for 1 hour, and phagocytosis of these fungal pathogens by CD14^+^ monocytes was analyzed by conventional flow cytometry and ImageStream. (**A**) Phagocytosis of *C. albicans* and *C. neoformans* by mock- and CMV-infected monocytes shown as overlay dot plots gated on calcein^+^ monocytes (left). Overlay histogram plots (middle) of the MFI of phycoerythrin (PE), the marker for phagosomal *C. albicans* and *C. neoformans*, from one representative experiment are shown. Intracellular, intraphagosomal *C. albicans* or *C. neoformans* was distinguished from extracellular, red-labeled fungi (blue) as pHrodo-red intensifies in fluorescence only when subject to an acidification in the phagosome. Layered histogram plots (right) show distinct separation of mock- and CMV-infected GFP^+^ monocytes by FITC but overlapping calcein plots. Results from a representative experiment are shown. (**B**) Whisker plots and before-and-after diagram of composite data from all biological and technical replicates from pHrodo phagocytosis assays (*n* = 10, *C. albicans P* = 0.002, *C. neoformans P* = 0.01, paired *t* test). (**C**) ImageStream analysis to visualize the phagocytic index of CMV-infected monocytes for *C. albicans* and *C. neoformans*. A panel of five representative mock- and CMV-infected monocytes subjected to a secondary challenge with pHrodo-labeled fungi confirms decreased phagocytic index of CMV-infected monocytes for both *C. albicans* and *C. neoformans*. Magnification, ×40. Scale bars, 10 μm. *P < 0.05 and **P < 0.01.

To understand whether the decreased expression of *CD36*, *MRC1*, and *CR3* altered the ability of CMV-infected monocytes to phagocytose fungal pathogens, we compared the capacity of CMV-infected monocytes to phagocytose *C. albicans* and *C. neoformans*—fungal pathogens that are recognized by these three surface fungal PRRs and cause clinically significant morbidity and mortality in transplant recipients (*24*, *27*). For this purpose, we measured uptake of pHrodo–phycoerythrin (PE)–labeled *C. albicans* and *C. neoformans* by mock- and CMV-infected PBMCs by flow cytometry. We quantified the phagocytic index, defined by quantitating the number of fungi ingested per phagocyte over 1 hour, by measuring the mean fluorescence intensity (MFI) of PE in CMV- and mock-infected monocytes (Fig. 4). CMV-infected monocytes had a significantly lower phagocytic index for both *C. albicans* and *C. neoformans* than did mock-infected cells (*P* = 0.002 and 0.01, respectively, paired *t* test) (Fig. 4, A and B). The decreased phagocytic index of CMV-infected monocytes was confirmed by ImageStream (Amnis), which showed reduced intracellular *C. albicans* and *C. neoformans* organisms in CMV-infected than in mock-infected monocytes (Fig. 4C). We also measured the percent phagocytosis of *C. albicans* and *C. neoformans*. While there was a significant difference in the phagocytic index of both *C. albicans* and *C. neoformans* in CMV- versus mock-infected monocytes (Fig. 4), the percentage of monocytes capable of phagocytosing *C. albicans* was not significantly different in CMV- and mock-infected monocytes; the percentage of monocytes capable of phagocytosing *C. neoformans* was significantly reduced (*P* = 0.01, paired *t* test) (fig. S5). These functional data demonstrate that CMV-infected monocytes have an impaired ability to phagocytose *C. albicans* and *C. neoformans*. These data also suggest that, similar to our previous findings in mouse macrophages (*27*), the decreased ability of CMV-infected monocytes to phagocytose fungal pathogens may be mediated through decreased surface expression of the class B scavenger receptor *CD36*, and this deficit may be further augmented by decreased expression of *MRC1* (*37*) and *CR3* (*24*).

### CMV infection of monocytes induces the inflammasome and pro-inflammatory molecules associated with allograft rejection

**Fig. 5:**
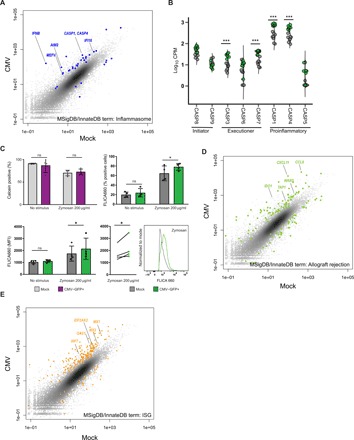
CMV infection of monocytes induces the expression of the *AIM2* and *IFI16* inflammasome and a diverse array of pro-inflammatory mediators associated with allograft rejection. Scatter plot showing all transcripts detected by RNA-Seq in mock- and CMV-infected CD14^+^/16^−^ monocytes. (**A**) Differentially expressed genes within curated lists related to inflammasome activation (blue) are highlighted. (**B**) Binary violin plots of mRNA expression of all caspases in mock- and CMV-infected monocytes. (**C**) Percentage of calcein-positive mock- and CMV-infected cells (top left) and caspase protein expression in mock- and CMV-infected monocytes 24 hours after infection with additional challenge with zymosan (*n* = 4; *P* = 0.03 and 0.02, respectively, two-way ANOVA with Bonferroni post-test), as measured by percentage of cells positive for FLICA (top right) and MFI of FLICA (bottom) with representative histogram (far right). (**D**) Differentially expressed genes within curated lists related to allograft rejection (green) and (**E**) ISGs (brown) are highlighted. *P* values (C) and adjusted FDR *P* values (B) are denoted as *P < 0.05, **P < 0.01, ***P < 0.001, and ****P < 0.0001. FLICA, fluorochrome inhibitor of caspase activity; ISG, interferon-stimulated genes.

In contrast to this “immunosuppressive” phenotype of CMV-infected monocytes with diminished capacity to phagocytose fungal pathogens, CMV induced the expression of several transcripts involved in inflammasome activation, a biological process enriched among up-regulated genes according to GSEA (Fig. 5A), suggesting a concomitant “immunostimulatory” phenotype. Genes encoding several innate immune sensors, which are components of the inflammasome complex, including the non-NLR, direct binder of cytosolic dsDNA *AIM2*, IFN-inducible *IFI16*, and pyrin-encoding *MEFV* (*38*–*40*), were significantly up-regulated in CMV-infected monocytes (logFC > 2.9, FDR < 1 × 10^−5^). Within the canonical NLR inflammasome family (*41*), *NLRP1* expression was decreased in CMV-infected monocytes (logFC = −1.8, FDR = 0.02) and the expression of *NLRP3*, *NLRC4*, and *NAIP* was not significantly changed (FDR > 0.1). There was up-regulation of *NLRC5* (logFC = 2.1, FDR = 0.0006), which has been shown to induce the expression of major histocompatibility complex (MHC) class I genes and type I IFNs (*42*), a pattern confirmed in our experimental model, where in CMV-infected monocytes, the expression of multiple MHC class I genes was up-regulated (logFC > 1.3, FDR < 0.04), and the expression of genes for many members of the *IFN*α family and *IFN*β*1* was also markedly induced (logFC > 7.4, FDR < 3.4 × 10^−24^). The expression of genes for caspases, endoproteases within the inflammasome that mediate both defense against pathogens and programmed cell death pathways, followed a very specific pattern in CMV-infected monocytes. While genes for initiators of apoptosis *CASP8* and *CASP9* and pro-apoptotic executioner *CASP3*, *CASP6*, and *CASP7* were either expressed at very low levels or not significantly changed, the pro-inflammatory, catalytic *CASP1*, *CASP4*, and *CASP5* genes were highly expressed and significantly up-regulated in CMV-infected monocytes (logFC ≥ 1.9, FDR = 0.003) (Fig. 5B). Moreover, there was also increased expression of the gene encoding for gasmerdin D (*GSDMD*), which, when cleaved by CASP1, CASP4, and CASP5, triggers pyroptosis (logFC = 1.8, FDR = 0.005) (*43*). To validate these findings at a functional level, we measured the percentage of mock- and CMV-infected monocytes that stained positively for fluorochrome inhibitor of caspase activity (FLICA), a molecule that binds irreversibly to the reactive cysteines of active caspases. While there was no significant difference in the expression of activated caspase proteins 24 hours after infection, there was a significant increase in both the proportion of CMV-infected monocytes with activated caspase protein expression and the MFI of caspase protein expression in CMV-infected monocytes after a subsequent challenge with the fungal surrogate zymosan [*P* = 0.02, two-way analysis of variance (ANOVA)] (Fig. 5C). These transcriptional and functional data suggest that CMV infection induces the expression of critical components of the inflammasome, including *AIM2*, *IFI16*, and *MEFV*, and primes monocytes to produce an exaggerated pro-inflammatory response by preferentially activating inflammatory caspases in response to an additional danger stimulus. These data also suggest that CMV infection of monocytes can mediate a pyroptotic environment without necessarily committing infected monocytes to undergo apoptosis or necrosis.

Having established that CMV-infected monocytes are capable of creating a pro-inflammatory, pyroptotic environment by priming of the inflammasome, we interrogated our RNA-Seq dataset to understand additional ways in which CMV infection of monocytes could mediate a pro-inflammatory milieu predisposing to allograft rejection, an important complication in SOT identified as an enriched biological process with a positive normalized enrichment score by GSEA. We found that several genes known to be associated with allograft rejection and dysfunction in SOT and GVHD in BMT were significantly up-regulated in CMV-infected monocytes. For example, *CCL8*, a chemokine shown to be a GVHD biomarker (*44*) and shown to predict CMV viral control in transplant recipients (*45*), was markedly up-regulated (logFC = 5.5, FDR = 1.1 × 10^−18^). We also found increased expression of genes for the triad of *CXCL11*, *CCL19*, and T cell co-stimulatory molecule *CD80* (B7-1), a recently described macrophage-associated signature predicting subclinical allograft injury in renal transplantation (logFC > 4, FDR < 1.5 × 10^−23^) (*46*). The combination of *CXCL11* and genes for the IFN-inducible enzymes *IDO1* and *WARS*, biomarkers for allograft rejection involved in tryptophan catabolism (*47*), was also markedly up-regulated in CMV-infected monocytes (logFC > 2.2, FDR < 0.0002) (Fig. 5D). In addition, the expression of several IFN-stimulated genes (ISGs) with well-described antiviral and immunomodulatory effects in the context of allograft rejection (*48*) was up-regulated in CMV-infected monocytes. This included increased expression of the nucleic acid sensing, antiviral-mediating oligoadenylate synthase (OAS) family of genes *OAS1*, *OAS2*, and *OAS3* (logFC > 2.3, FDR < 0.0002), type I and III IFN-induced antiviral guanosine triphosphatase (GTPase) genes *MX1* and *MX2* (logFC > 2.7, FDR < 8.1 × 10^−6^), the protein kinase *EIF2AK2* gene (logFC = 2, FDR < 0.001), and *IRF7* (logFC = 3.3, FDR < 3.8 × 10^−8^), an IFN-stimulated transcription factor shown to have an important role in controlling viral replication and mediating inflammation associated with herpes virus infections (Fig. 5E) (*48*–*50*). Together, these data highlight specific pathways through which CMV-mediated modulation of monocytes may mediate the indirect effects of allograft rejection and GVHD in transplantation (table S4).

To determine whether this pattern of expression of genes involved in viral and nonviral pathogen recognition, inflammasome activation, and allograft rejection required active CMV gene expression, we performed RNA-Seq on CD14^+^/16^−^ monocytes infected with ultraviolet (UV)–inactivated CMV. PBMCs isolated from CMV IgG-negative healthy donors were infected with TB40/E-5 subject to 10 (UV^TC10^) and 60 (UV^TC60^) of direct UV light; mock and live-TB40/E-5 infections were simultaneously performed to allow direct comparison of transcriptional profiles elicited by all three conditions (table S5). These conditions were established based on an experiment testing different methods of inactivation (fig. S6). Sixty minutes of exposure to UV light resulted in greater than 10-fold reduction in GFP expression, a surrogate for TB40/E-5 infection, in UV-infected versus live virus–infected CD14^+^/16^−^ monocytes, while 10 min of exposure resulted in twofold decrease in GFP expression (table S6). In CD14^+^/16^−^ monocytes infected with UV-inactivated CMV, there was up-regulation of expression of viral PRR transcripts within the RLR, DAI, and NOD families and decreased expression of fungal PRRs *CD14*, *MRC1*, *STAB1*, and *ITGAM/ITGB2*, similar to monocytes infected with live, replication-competent CMV. There was no significant change in the expression of *CD36* in monocytes exposed to UV-inactivated virus. There was also increased mRNA expression of GVHD and rejection biomarkers *CCL8*, *CXCL11*, *IDO1*, *WARS*, and ISGs, as well as inflammasome-associated transcripts *AIM2*, *IFI16*, and *GSDMD*, although there was no significant increase in the expression in *CASP1*, *CASP4*, or *CASP5* in monocytes infected with UV-inactivated CMV (table S7). Collectively, these data suggest that the concurrent immunosuppressive and immunostimulatory transcriptional profiles elicited in monocytes by CMV infection can occur even when viral expression is greatly reduced, although there may be individual deficits or states of activation in scavenger receptor and inflammasome pathways, which are induced or enhanced by active CMV gene expression and/or replication.

### Single-cell RNA-Seq of monocytes infected with CMV in vitro and in vivo shows up-regulation of viral PRRs and ISGs but heterogenous expression of fungal PRRs

**Fig. 6:**
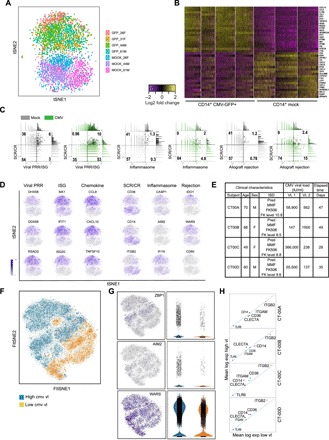
scRNA-Seq demonstrates that CMV-infected monocytes uniformly express viral PRRs, IFN-induced cytokines, and pro-inflammatory chemokines but maintain a heterogenous pattern of expression of scavenger receptors, complement receptor 3, inflammasome activation, and non-chemokine biomarkers of allograft rejection. (**A**) Single mock CD14^+^ and CMV-infected CD14^+^GFP^+^ monocytes cluster in distinct populations by tSNE. (**B**) Heat map with hierarchical clustering demonstrating the top 20 up-regulated and down-regulated transcripts in CMV-infected monocytes compared to mock. (**C**) SeqGeq dot plot demonstrating the combined expression of families of transcripts, including scavenger receptor/complement receptor (Σ *CD36*, *CD14*, *STAB1*, and *ITGB2*), viral PRRs (Σ *MX1*, *MX2*, *DDX58*, and *DHX58*), inflammasome (Σ *CASP1*, *CASP4*, *AIM2*, and *IF16*), and allograft rejection (Σ *IDO1*, *WARS*, *TAP1*, and *CD80*) in single cells from mock- and CMV-infected monocytes. (**D**) tSNE plots comparing expression of *DDX58*, *DHX58*, *RSAD2*, *MX1*, *IFIT1*, *ISG20*, *CCL8*, *CXCL10*, and *TNFSF10* to expression of *CD36*, *CD14*, *ITGB2*, *CASP1*, *AIM2*, *IFI16*, *IDO1*, *WARS*, and *CD80* in CMV-infected monocytes. Single-cell expression is represented on gray-purple scale. (**E**) Clinical characteristics of four cardiac transplant recipients with detectable peripheral CMV viremia. (**F**) CD14^+^ monocytes obtained from heart transplant recipients during periods of high and low CMV viremia cluster in distinct populations by FItSNE. (**G**) Violin plots demonstrating expression of viral PRR *ZBP1*, inflammasome gene *AIM2*, and allograft rejection–associated genes *WARS* in all four transplant recipients and (**H**) scatter plots showing changes in expression of PRRs *CD36*, *CD14*, *ITGAM/ITGB2*, *CLEC7A*, and *TLR6* from individual heart transplant recipients. Genes with significant expression changes by Welch’s *t* test, Fisher’s exact test, and Mann-Whitney *U* test with Benjamini-Hochberg procedure applied are shown in capital letters; genes depicted in lowercase italics did not meet criteria for significant change in expression. PRR, pathogen recognition receptor. SCR, scavenger receptor; VL, viral load.

Having demonstrated that CMV infection of CD14^+^/16^−^ monocytes mediates deficits in phagocytosis (immunosuppressive phenotype) and induction of the inflammasome and pro-inflammatory mediators of allograft rejection ( immunostimulatory phenotype), with similar results in CD14^+^/16^+^ monocytes (table S4), we used single-cell RNA sequencing (scRNA-Seq) to understand the heterogeneity of these responses in CMV-infected monocytes. First, we evaluated the expression of genes identified as putative mediators of CMV-associated indirect effects in transplantation across a total of 2179 CD14^+^GFP^+^ CMV-infected and 2048 CD14^+^ mock-infected monocytes from our in vitro model. CMV- and mock-infected monocytes clustered in distinct populations by PCA and *t*-distributed stochastic neighbor embedding (tSNE), and the expression of key individual genes identified in bulk RNA-Seq analysis followed the same patterns in scRNA-Seq (Fig. 6A). This included increased expression of genes for viral PRRs (*DDX58*, *DHX58*, and *IFIH1*) and ISGs (*MX1* and *MX2*) and decreased expression of genes for scavenger receptors (*CD14*, *CD36*, and *STABILIN-1*) and complement-receptor 3 component (*ITGB2*). *CCL8*, *CXCL10*, *CXCL11*, *TAP1*, *WARS*, *IDO1*, and *ISG20*, transcripts associated with allograft rejection and significantly up-regulated in the bulk RNA-Seq dataset, were also up-regulated in scRNA-Seq (Fig. 6B). We plotted the combined expression of nonviral PRR (Σ *CD36*, *CD14*, *STAB1*, and *ITGB2*), viral PRR/ISG (Σ *MX1*, *MX2*, *DDX58*, and *DHX58*), inflammasome (Σ *CASP1*, *CASP4*, *AIM2*, and *IFI16*), and allograft rejection (Σ *IDO1*, *WARS*, *TAP1*, and *CD80*) transcripts in mock- and CMV-infected monocytes. In CMV-infected monocytes, we found that the expression of viral PRRs/ISGs increased by sevenfold, inflammasome activation by fourfold, and allograft rejection by eightfold relative to mock-infected cells; in contrast, nonviral PRR gene expression decreased by sixfold (Fig. 6C). While CMV-infected monocytes had uniformly increased expression of viral PRRs, ISGs, and pro-inflammatory chemokine genes across all cells, only a proportion of CMV-infected monocytes had increased expression of *CASP1*, *AIM2*, *IFI16*, *IDO1*, *WARS*, and *CD80* and decreased expression of *CD36*, *CD14*, and *ITGB2* (Fig. 6D).

To determine whether this pattern of dual immunosuppressive and immunostimulatory phenotypes of CMV-infected monocytes detected in our in vitro model also existed in vivo, we performed scRNA-Seq on monocytes isolated from four heart transplant recipients, maintained on the same combination immunosuppressive regimen of prednisone, mycophenolate mofetil, and tacrolimus (level, 8.5 to 10.8). For patients CT00A, CT00C, and CT00D, PBMCs were obtained within 48 hours of polymerase chain reaction (PCR)–based detection of the CMV viral load as reported by the Massachusetts General Hospital (MGH) Microbiology laboratory for clinical care. In these three subjects, we defined “high” and “low” viral load specimens, with high CMV viral load ranging from 55,000 to 360,000 IU/ml and low viral load ranging from near undetectable viral load <137 to 562 IU/ml. For study subject CT00B, PBMCs were obtained within 48 hours of detecting high CMV viral load of 1500 IU/ml copies in the setting of reactivation of CMV, which was an increase from viral load <137 IU/ml. All four patients were maintained on intravenous ganciclovir or oral valganciclovir to treat CMV at each time point of PBMC isolation, without interruption for the duration of time between selected time points (Fig. 6E). To further control for the effect of immunosuppressive medications and antiviral therapy on the transcriptome of immune cells, each subject served as his/her own control in a pairwise analysis. Calcein^+^CD45^+^14^+^ monocytes were isolated by flow cytometry from PBMCs isolated from whole blood during routine monitoring of response antiviral therapy, with immune suppression medication regimen otherwise unchanged. Fast Fourier transform–accelerated interpolation-based tSNE (FItSNE) plots demonstrated clustering of monocytes isolated during high and low/undetectable CMV viremia (Fig. 6F), and differential gene expression analysis comparing transcriptomes of monocytes isolated during periods of high CMV viral load as compared to low/undetectable CMV viral load revealed significant up-regulation of expression of genes involved in RLR and DAI families of viral PRRs, allograft rejection, and ISGs in all transplant recipients (Fig. 6G), similar to our findings in our in vitro model (table S8). While all four subjects had up-regulation of expression of *AIM2* (Fig. 6G), and three of the four subjects had increased expression of other inflammasome-associated transcripts, one subject had significantly decreased expression of *IFI16*, *MEFV*, *CASP1*, and *CASP4*. Significant changes in the expression of *CD36*, *CD14*, *ITGB2/ITGAM*, and *TLR6* were also identified in unbiased differential expression analysis of monocyte transcriptomes in these heart transplant recipients. However, there was heterogeneity in the expression of these fungal PRR genes in vivo. In two of the four subjects (CMV-CT00A and CT00B), the expression of *CD14*, *CD36*, *ITGB2*, *TLR6*, and *STAB1* was down-regulated in monocytes isolated during periods of high CMV viremia, and in the other two subjects (CMV-CT00C and CT00D), there was significant up-regulation of the expression of these scavenger receptors and complement receptor 3 in the setting of high CMV viral load (Fig. 6H and table S8). These data reinforce the importance of the dysregulated pathways identified from our in vitro model of CMV infection of monocytes. Moreover, both the in vitro and in vivo scRNA-Seq data demonstrate that there are certain transcriptional changes, namely, increased expression of viral PRRs, ISGs, and allograft rejection–associated genes, that occur for all patients during periods of lytic CMV infection. In contrast, there is greater heterogeneity in the expression patterns of genes involved in the activation of the inflammasome and recognition of fungal PRRs in the setting of lytic CMV infection, suggesting that while CMV is one important modulator of these innate immune pathways, there may be additional factors that co-regulate these pathways in the setting of transplantation (table S9).

## DISCUSSION

In this study, we introduce a framework for understanding the monocyte-CMV host-pathogen interface in human disease, with a focus on illuminating the biological mechanisms underpinning indirect effects in transplantation. Our analysis of transcripts differentially expressed in CMV-infected monocytes identify innate immune pathways modulated by CMV infection that have not been collectively previously described. These include pathways regulating phagocytosis of fungal pathogens in the context of diminished expression of fungal PRRs *CD36*, MRC1, and complement receptor 3; induction of *AIM2*, *IFI16*, and pyrin inflammasomes; and up-regulation of mediators of chronic inflammation, which could be biomarkers for allograft dysfunction, rejection, and GVHD in transplantation.

Our in vitro model of CMV infection recapitulates the clinical scenario of a CMV-seronegative recipient (R^−^) receiving an organ from a CMV-exposed donor (D^+^), in which there is innate and adaptive immune cross-talk and high risk for developing indirect effects in the setting of CMV discordance while eliminating the confounding effects of variable pharmacologic immunosuppression and viral strain in the transcriptional and functional innate immune landscape. By interrogating transcriptional and functional changes induced in monocytes by CMV infection 24 hours after infection in vitro, we modeled and defined a host-pathogen interaction that would have been very difficult to detect in vivo. In this respect, our evaluation of the monocyte-CMV host-pathogen interface in vitro and in vivo in solid organ transplant recipients complements each other, providing snapshots of how CMV can modulate the monocyte transcriptome at important immunologic and virologic time points, including during the very first steps after a CMV “naïve” monocyte is exposed to virus; during active, ongoing viral replication during lytic CMV infection; and during development of CMV latency with reduction in CMV viremia.

The down-regulation of the surface expression of CD36 in human monocytes in the setting of early CMV infection is particularly noteworthy, as CD36 has been independently identified in other studies evaluating the innate immune system–CMV host-pathogen interface in different CMV infection “states” (*51*, *52*). This scavenger receptor has been shown not only to be a mediator of antifungal immunity (*27*) but also to promote tolerance to host antigens in transplantation by facilitating transfer of medullary thymic epithelial cell–derived antigens to dendritic cells (*53*). Thus, CMV-mediated down-regulation of CD36 could simultaneously diminish the capacity of the innate immune system to recognize fungal pathogens and promote a proinflammatory environment, contributing to allograft rejection and GVHD; this suggests that CD36 could be a master regulator of indirect effects in transplantation. Similarly, while decreased CR3 expression leads to decreased phagocytosis, because CR3 is also a negative regulator of TLR signaling (*54*), diminished CR3 expression could itself lead to a hyper-responsiveness to TLR-mediated responses to virus, thus increasing IFN and pro-inflammatory cytokine production, further predisposing to allograft rejection. Further investigation of human genomic data to assess whether single-nucleotide polymorphisms (SNPs) in CD36 and CR3 could predict CMV-related outcomes in transplantation will advance our understanding of CMV-related outcomes in transplantation.

Our study suggests that the innate immune system may be a major contributor to the biology of rejection and GVHD, links many of these biomarkers to CMV-mediated changes in innate immunity, and allows the discovery of new, putative CMV-associated biomarkers of allograft rejection and GVHD. For example, in a manual interrogation of our in vitro dataset, we found marked up-regulation of expression of genes encoding a wide variety of pro-inflammatory molecules, which have been described as mediating inflammatory states in human disease but not implicated in allograft rejection, including chemokine genes *CCL18* and *CXCL13*; membrane-associated protein sheddase gene *ADAM19* (*55*); *NINJ1*, a gene encoding an adhesion molecule induced in the setting of chronic inflammatory disease states including neuroinflammation and pulmonary fibrosis (*56*); and *CD38*, encoding a marker of chronic immune activation in HIV-1 infection (*57*). The roles of these genes in mediating allograft rejection and GVHD are not known, but we have laid the groundwork for continued study.

Our data show that CMV infection alone is enough to alter the host innate immune response, paralyzing its ability to recognize and phagocytose fungal pathogens, adding to the growing body of literature demonstrating that a viral infection can render the innate immune response defective or maladaptive in the context of additional concomitant bacterial or fungal infection (*58*). Moreover, we define CMV-induced transcriptional and functional changes during early CMV infection, which not only render human monocytes deficient in phagocytosing fungal pathogens but also are capable of creating a pyroptotic, pro-inflammatory milieu. These observations have important clinical implications, as many of the effects of CMV on innate immune function in transplantation may be occurring after attachment of viral particles, but before viral gene expression, a conclusion supported by our data showing that the changes in expression of viral PRRs, fungal PRRs, inflammasome activation, and allograft rejection also occur, with UV-inactivated CMV exhibiting impaired viral gene transcription and viral replication. These data suggest that inhibitors of stages of infection during this window, such as viral entry, may have a unique role in the prophylaxis and treatment of CMV infection in the immunocompromised host to mitigate the indirect effects of CMV in transplantation (current approved drugs act during DNA synthesis or later in infection).

This study demonstrates how CMV can modulate monocyte transcriptional and functional phenotypes to be both immunosuppressive and immunostimulatory, providing insight into CMV status and transplant-associated disease risk in the immunocompromised host. This concept is supported by transcriptional profiling of monocytes from heart transplant recipients with CMV viremia, in whom important genes in pathways identified from our in vitro bulk and scRNA-Seq models were also found to be dysregulated in vivo. Our finding of uniform induction of expression of ISGs, and genes involved in viral recognition and allograft rejection but heterogeneous expression of genes involved in fungal pathogen recognition and inflammasome activation, provides a compelling glimpse into ways in which CMV modulates the innate immune response in solid organ transplant recipients. This observed heterogeneity is consistent with variability observed in the clinical setting between patients and may reflect differential effects of various strains of CMV with different tropisms for monocytes, variation in duration and response to antiviral therapy, individual responses to comparable immunosuppressive therapies, or genetic differences in the control of the immune response to CMV (*59*). Our observations underscore the importance of pursuing translational, “omics”-based studies of both the innate and adaptive immune system to better understand CMV-associated transplantation-related outcomes.

## MATERIALS AND METHODS

### Virus culture, propagation, and purification

To reconstitute infectious human CMV derived from a single bacterial artificial chromosome (BAC) clone, BAC containing the human CMV TB40/E-5 genome and GFP under control of an SV40 promotor (gift from F. Goodrum, University of Arizona) was used to electroporate human foreskin fibroblasts (HFFs; American Type Culture Collection, CRL-1684), as described previously (*60*). To generate virus stocks, HFFs were cultured in Dulbecco’s modified Eagle’s medium (DMEM), 10% heat-inactivated fetal bovine serum (hiFBS), penicillin (100 IU/ml), and streptomycin (100 μg/ml) (Corning) in T225 flasks (Corning) and once at 90% confluence, infected with TB40/E-5 at a multiplicity of infection (MOI) of 0.01, and cultured for an additional 10 to 14 days in DMEM, penicillin/streptomycin, and 5% FBS. Supernatants from culture flasks were harvested and centrifuged at 1600 rpm for 10 min to pellet HFF cell debris. Subsequently, supernatant clear of HFF debris was layered on a 20% sucrose gradient made in phosphate-buffered saline (PBS) without calcium/magnesium and subject to ultracentrifugation at 25,000 rpm at 4°C for 60 min. Viral pellets were then reconstituted in DMEM, penicillin/streptomycin, and 10% hiFBS and stored in single-use aliquots in liquid nitrogen. Titration of virus was conducted by infecting HFFs seeded in 24-well plates (1 × 10^5^ cells per well) with serial dilutions of virus stock. After 2 hours, the inocula were replaced with DMEM containing methylcellulose (6 g/liter), 5% FBS, and 1% penicillin/streptomycin. After 14 days, cell monolayers were fixed with a mixture of glacial acetic acid and methanol (at a 1:2 ratio) and stained with crystal violet, and plaques were counted with a dissecting microscope. The titers obtained represent an average of duplicate samples. TB40/E-5 used to infect monocytes subsequently used for RNA-Seq were passage 5 and lower; all TB40/E-5 used in functional assays were passage 7 and lower. HFF cultures were periodically tested for mycoplasma contamination and found to be negative.

### UV inactivation of CMV

In an initial experiment, TB40/E-5 was subject to UV inactivation by two methods: exposure to UV lamp in tissue culture hood at a distance of 6 inches for 60 min (UV^TC^) (*61*) or by applying pulse (360 mJ/cm^2^) via the Stratagene Stratalinker (UV^SL^) (*62*) as previously described. To compare the efficiency of these two methods of UV inactivation, quantitative reverse transcription (qRT)–PCR, Western blot, immunohistochemistry, and flow cytometric measurement of *GFP* and *IE1* expression were conducted on HFF infected with UV-inactivated CMV and compared to HFF concurrently infected with non–UV-inactivated TB40/E-5 (Live) and mock infection. For GFP and IE1 qRT-PCR, total RNA was purified from infected cells using the AllPrep DNA/RNA Mini Kit (Qiagen), and reverse transcription was carried out with the QuantiTect Reverse Transcription Kit (Qiagen) following the manufacturer’s protocol except for the following: 3 nM gene-specific reverse primers were used in 10-μl total reaction volume, and for *IE1*, the reactions were incubated at 42°C for 45 min rather than 15 min. Real-time PCR was performed on complementary DNA (cDNA) using SYBR Green PCR Master Mix and the StepOnePlus Real-Time PCR System (Thermo Fisher Scientific). *GFP* and *IE1* RNA levels were normalized to human glyceraldehyde-3-phosphate dehydrogenase (hGAPDH) levels. Relative RNA copy numbers represent the mean of duplicate wells and were determined from the standard curves of dilution series. DNA contamination was not detected in any samples. A standard curve for enhanced GFP (EGFP) qRT-PCR was generated by serially diluting purified total RNA obtained from 293T cells transfected with a plasmid expressing EGFP into total RNA from untransfected 293T cells. The standards for *IE1* and *hGAPDH* were generated by serially diluting homogenates of CMV-infected 293T cells. Primers for reverse transcription and PCR were as follows: hGAPDH, GAAGGTCGGAGTCAACGGATT (forward) and GCCTTGACGGTGCCATGGAA (reverse) [Integrated DNA Technologies (IDT)] (*63*); EGFP, GAACCGCATCGAGCTGAA (forward) and TGCTTGTCGGCCATGATATAG (reverse) (Invitrogen); and IE1 (UL123), ACGAGAACCCCGAAAAAGATG (forward) and CGCCAGTGAATTTCTCTTC (reverse) (IDT) (*64*). Western blots to detect GFP and UL84 were performed using equal parts protein lysates in 3× boiling mix [30% stacking gel buffer (SGB), 30% glycerol, 6.5% SDS powder, 9 M urea, 100 mM dithiothreitol, and bromophenol blue] from UV-inactivated TB40/E-5, Live-TB40E/5, and mock-infected HFF at MOI 1 at 24 and 72 hours after infection. Protein was transferred to nitrocellulose membrane (Amersham), and blocking was performed in non-fat milk. Rabbit anti-GFP (1:1000; Sigma-Aldrich, G1544), mouse anti–β-actin (1:1000; Sigma-Aldrich, A5441), anti-CMV UL84 (1:1000; Virusys, CA144), goat anti-mouse IgG/human adsorbed horseradish peroxidase (HRP) (1:1000; Biotech, 1030-05), and goat anti-rabbit IgG-HRP (1:2000; Biotech, 4030-05) were used for protein detection. Subsequent experiments used the UV^TC^ method for 10 or 60 min, with analysis by RNA-Seq.

### CMV infection of PBMCs in vitro

Red blood cell–depleted buffy coats, whole blood, and serum were obtained from immunocompetent donors from the MGH blood bank and Stem Express in accordance with Partners Institutional Review Board (IRB)–approved informed consent. CMV IgG testing was performed on whole serum [Zeus Scientific enzyme-linked immunosorbent assay (ELISA)], and only CMV IgG-negative blood donors were used for all subsequent experiments. Buffy coats were centrifuged over Histopaque 1077 (Sigma-Aldrich) gradient for 30 min at 400*g*, and the mononuclear layer was isolated and washed with PBS. Isolated PBMCs were then resuspended in Roswell Park Memorial Institute medium (RPMI) supplemented with l-glutamine, 10% autologous human serum from the donor, penicillin (100 IU/ml), and streptomycin (100 μg/ml) (Corning) and seeded at 4 × 10^6^ PBMCs (approximately 4 × 10^5^ monocytes) per well in 1.0 ml of complete medium in a 12-well plate. Subsequently, 4 × 10^5^ particle-forming units (PFU) TB40/E-5 resuspended in 125 μl of complete RPMI was added to each well. For mock-infected plates, 125 μl of complete RPMI without virus was added to each well containing 4 × 10^6^ PBMCs. Plates were incubated for 30 min at 37°C and then centrifuged at 900*g* for 60 min at 37°C. Following this “spinfection,” cells were cultured in the dark at 37°C in a 5% CO_2_ humidified incubator for an additional 24 hours.

PBMCs isolated from CMV IgG-negative healthy donors were infected with TB40/E-5 subject to 10 min (UV^TC10^) and 60 min (UV^TC60^) of direct UV light; additionally, mock and live-TB40/E-5 infections were simultaneously performed to allow direct comparison of transcriptional profiles elicited by each infection state. For the UV inactivation experiments, aliquots of pre-titered TB40/E-5 were pooled, with equal parts of the pool subject to 0, 10, and 60 min of UV light before use for spinfection as detailed above.

### PBMCs from CMV-infected solid organ transplant recipients

Samples of 10 to 20 cm^3^ of whole blood were obtained from heart transplant recipients during periods of documented CMV viremia in accordance with Partners IRB-approved informed consent. CMV viral loads from transplant recipients were measured as clinically indicated on the Roche Cobas AmpliPrep platform and reported by the MGH Microbiology laboratory as international units (IU/ml). Blood was centrifuged over Histopaque 1077 for 30 min at 400*g*, and the mononuclear layer was isolated and washed with PBS. Frozen aliquots of PBMCs were stored at −80°C in 90% FBS/10% dimethyl sulfoxide. Individual vials of PBMCs were thawed on ice and stained with calcein-violet AM, CD45^FITC^, and CD14^APC^ (BioLegend), and calcein^+^CD45^+^14^+^ monocytes were sorted from PBMCs isolated on the BD Fusion. Isolated monocytes were centrifuged at 250*g* for 10 min at 4°C, resuspended in 0.1% bovine serum albumin (BSA)/PBS, and vortexed to ensure a single-cell suspension. Trypan blue staining was done to confirm that cells entering the scRNA-Seq workflow had greater than 95% viability.

### Immunohistochemistry and microscopy

Individual 1.5 thickness circular glass slides were sterilized and placed in a 24-well tissue culture plate. Phorbol 12-myristate 13-acetate (PMA; 2 × 10^6^)–stimulated THP-1 cells per well were added to each slide, and 2 × 10^5^ PFU TB40/E-5 were added to each well. The plates were incubated for 30 min at 37°C and then centrifuged at 900*g* for 60 min at 37°C. Following this spinfection, cells were cultured in the dark at 37°C in a 5% CO_2_ humidified incubator for an additional 24 hours, after which the supernatant was gently aspirated, slides were fixed with 4% paraformaldehyde (PFA) for 10 min, washed with PBS two times, and then permeabilized with 0.1% Triton X-100 for 10 min at room temperature. CMV IE1/2 antibody (Virus-system) was added at 1:200 and allowed to incubate overnight at 4°C. Slides were subsequently washed with PBS and stained with goat anti-mouse Alexa 647 secondary. GFP signal was augmented by adding fluorescein isothiocyanate (FITC) anti-GFP. After a 1-hour incubation with secondary antibodies at room temperature, slides were washed and stained with 500 nM 4′,6-diamidino-2-phenylindole (DAPI). All slides were imaged on a Zeiss LSM 700 confocal microscope.

### Cell staining and flow cytometry

Mock-infected and TB40/E-5 GFP-infected monocytes were stained with calcein-violet AM, CD3/19/20/56^APC^, CD14^AF700^, and CD16^PE^, and circulating dendritic cells were additionally stained using HLA-DR^APC-Cy7^, CD11c^PECy7^, and CD123^BV605^ (BioLegend). Calcein^+^, CD3/19/20/56^−^, CD14^+^/16^−^, and CD14^+^/16^+^ monocytes (mock) and CD14^+^/16^+^/GFP^+^, CD14^+^/16^−^/GFP^+^, and CD14^+^/16^+^/GFP^+^ (TB40/E-5 infected) were sorted using flow cytometry (BD Fusion, 100-μm nozzle) into PBS and used for downstream bulk RNA-Seq. Calcein^+^, CD3/19/20/56^−^, and CD14^+^ (mock) and CD14^+^/GFP^+^ (TB40/E-5 infected) cells were sorted and made into single-cell suspensions for scRNA-Seq using the SureCell platform. PBMCs isolated from whole blood from heart transplant recipients were stained with calcein-violet AM, CD45^FITC^, and CD14^APC^ and made into single-cell suspensions for scRNA-Seq using the inDrop platform.

### Measuring expression of human scavenger receptors

Mock- and TB40/E-5–infected PBMCs were harvested after infection as detailed above and resuspended in PBS with 1% hiFBS. Following incubation on ice for 15 min in the presence of human Fc block (1:100; eBioscience), PBMCs were stained for 30 min on ice with CD36^APC^ (1:100; BioLegend) or CD14^APC^ (1:100; BioLegend), washed in PBS two times, and subsequently stained with calcein^BV421^ (1:500) for 15 min at room temperature, washed two times, and then analyzed with BD Fusion flow cytometer and FlowJo 10.1 software. All biological and technical replicates were included in analysis.

### Inflammasome assay

Mock- and TB40/E-5–infected PBMCs were harvested after infection as detailed above and resuspended in PBS with 1% hiFBS. PBMCs were incubated with FLICA660 (ImmunoChemistry Technologies, FLICA polycaspase assay) at 1:500 for 60 min in the dark at 37°C in a 5% CO_2_ humidified incubator. PBMCs were subsequently washed with PBS two times and resuspended in PBS with 1% hiFBS, and unbound FLICA was allowed to permeate out of the cells by incubating at 37°C in a 5% CO_2_ humidified incubator for an additional 15 min. PBMCs were subsequently resuspended in PBS without FBS and stained with calcein (1:500) for 20 min at room temperature and washed with PBS two times. The stained cells were analyzed with a BD Fusion flow cytometer and FlowJo 10.1 software. Caspase activity was measured in mock- and TB40/E-5–infected PBMCs 24 hours after infection without any additional stimulus and after an additional 4 hours of incubation with zymosan (200 μg/ml). The percentage of mock-infected and TB40E/5 GFP^+^ CMV-infected monocytes with activated caspases was calculated by taking the number of positive FLICA660 events (Q2 for mock and Q1 for TB40-GFP^+^) and dividing by the total number of monocytes analyzed (Q2 + Q3 for mock and Q1 + Q4 for TB40-GFP^+^). All biological and technical replicates were included in analysis.

### Phagocytosis assay

*C. albicans* and *C. neoformans* were streaked on yeast extract peptone dextrose (YDP) agar; individual *C. albicans* colonies were subsequently grown in YDP medium at 35°C, and individual *C. neoformans* colonies were grown at 25°C. *C. albicans* and *C. neoformans* were then heat-killed for 1 hour at 50°C and frozen back in single-use aliquots at −20°C. *C. albicans* and *C. neoformans* were fluorescently labeled using pHrodo-red (Invitrogen) as per the manufacturer’s instructions and resuspended in RPMI, penicillin/streptomycin, and 10% hiFBS. pHrodo-red–labeled *C. albicans* and *C. neoformans* were then added to mock- and TB40/E-5–infected mixed PBMC cultures at a ratio of 5:1 (yeast to monocyte) and were incubated in the dark at 37°C in a 5% CO_2_ humidified incubator for 1 hour. Stained cells were analyzed with a BD Fusion flow cytometer, and the data were analyzed with FlowJo10.1 software. All biological and technical replicates were included in analysis.

### ImageStream analysis

TB40/E-GFP–infected PBMCs incubated with pHrodo-red–labeled *C. albicans* and *C. neoformans* were processed and stained as detailed above. After staining, cells were fixed with 2% PFA and then stained with DAPI to a final concentration of 1 mg/ml. Flow imaging was done using Amnis ImageStream mkII Imaging Flow Cytometer, and data were analyzed using the IDEAS software.

### RNA isolation and bulk RNA-Seq

Mock-infected and TB40/E-5–infected mixed PBMCs were stained with calcein-violet AM, CD3/19/20/56^APC^, CD14^AF700^, and CD16^PE^, and calcein^+^, CD3/19/20/56^−^/CD14^+^16^−^, and CD14^+^16^+^ cells were sorted on the BD Fusion. Isolated cells were centrifuged at 350*g* for 10 min at 4°C and then resuspended in RLT lysis buffer (Qiagen) with β-mercaptoethanol. Resuspended cells were subjected to DNA shredding via the QiaShredder, and genomic DNA (gDNA) was eliminated via the gDNA eliminator column (Qiagen). To further ensure that there was no carryover of gDNA into RNA isolation, an on-column deoxyribonuclease digestion (Qiagen) was also performed. Isolated RNA was quantified and assessed for quality on a Bioanalyzer (Agilent); all RNA used for next-generation library preparation had RNA integrity number values of >9. Library construction was done using the 3′ QuantSeq Kit (Lexogen), and sequencing was completed on a Mini-Seq Illumina sequencer in our laboratory at the Center for Immunology and Inflammatory Diseases (CIID) at MGH.

### Single-cell RNA-Seq

Mock-infected and TB40/E-5–infected mixed PBMCs from the in vitro model were stained with calcein-violet AM, CD3/19/20/56^APC^, and CD14^AF700^, and calcein^+^ and CD3/19/20/56^−^/CD14^+^ cells were sorted on the BD Fusion. Isolated cells were centrifuged at 350*g* for 10 min at 4°C and then resuspended in 0.1% BSA/PBS to a final concentration of 3000 cells/μl and vortexed to ensure a single-cell suspension. Trypan blue staining was done to confirm that cells entering the scRNA-Seq workflow had greater than 95% viability. For mock- and CMV-infected monocytes isolated from in vitro infections, single cells were then encapsulated into oil droplets using the Bio-Rad ddSEQ Single-Cell Isolator. The Illumina Bio-Rad SureCell WTA 3′ Library Prep kit PBMC protocol was used to generate the cDNA libraries from RNA released from encapsulated cells during cell lysis and oil droplet disruption. Final libraries were quantitated and evaluated for quality control on the Agilent bioanalyzer in the CIID and MGH. scRNA-Seq libraries from calcein^+^CD45^+^14^+^ monocytes obtained from cardiac transplant recipients were constructed using the inDrops-Seq methodology in conjunction with the Harvard Medical School Single Cell Core as previously described (*65*). All scRNA-Seq libraries were sequenced on a Next-Seq Illumina sequencer at the Molecular Biology Core Facilities at Dana-Farber Cancer Institute.

### RNA-Seq analysis

STAR aligner (*66*) was used to map sequencing reads to transcripts in the human hg19 reference genome. Read counts for individual transcripts were produced with HTSeq-count (*67*), followed by the estimation of expression values count per million (CPM) reads and detection of differentially expressed transcripts using EdgeR (*68*). Expression heat map showed fold change difference compared to average expression across all samples. Differentially expressed genes were defined by at least twofold change with FDR less than 0.001. Sequences generated from RNA-Seq libraries that did not align to human hg19 reference genome were aligned to the genomic sequence of CMV virus (TB40/E BAC, GenBank accession no. EF999921.1) and the EGFP sequence. Two TB40/E-5–infected specimens in the CD14^+^/16^−^ monocyte subset had a higher percentage of viral and GFP reads compared to other analyzed specimens (fig. S2; 2.5% versus 0.001 to 0.100% viral reads and 0.23 to 0.32% versus 0.01 to 0.05% GFP reads, respectively) and were not included in the final analysis. We performed GSEA of expression changes by applying the GSEA tool against the hallmark gene sets in MSigDB (*19*) and tested functional enrichment of up- and down-regulated genes in InnateDB (*20*) pathway databases. Individual pathways significantly enriched generated from the Hallmark (normalized enrichment scores spanning −2.0 to 4.0, adjusted FDR value of less than or equal to 0.1) and InnateDB (*P* value less than or equal to 0.05) were then collated based on similarity of key terms and assigned a summary term to denote the sigma of these individual pathways.

For in vitro scRNA-Seq data, filtering and tSNE implementation were analyzed by SEURAT (*69*). Cells with gene number >200 and genes detected in >3 cells were used for the cell type clustering analysis. PCA was performed using the top 5000 most variable genes, and PCs 1 to 20 were then used as input for tSNE to generate a two-dimensional (2D) nonlinear embedding of the cells. To determine the marker genes, we identified top genes enriched in each cluster using nonparametric binomial test between mock and CMV cells. scRNA-Seq data analysis was also performed in SeqGeq version 1.5.0 (FlowJo).

For in vivo scRNA-Seq data from heart transplant recipients, we generated gene count matrices using scumi v0.1.0 (*70*) and performed downstream analysis using Pegasus (*71*) v0.15.0. In particular, we selected only high-quality cells using the following criteria: (i) number of expressed genes between 500 and 6000 and (ii) percentage of unique molecular identifiers (UMIs) from mitochondrial genes <10%. We then normalized UMI counts for each cell into expression levels in TP100K based on only genes that were expressed in at least 0.05% of all selected cells and then transformed expression levels into log space by log(*TP*100*K* + 1). Next, we selected the top 2000 highly variable genes (HVGs), computed the top 50 PCs from the HVGs, and generated the 2D FItSNE (*72*) embedding based on the PC space. We applied the above analyses to the merged dataset comprising all four patients to generate Fig. 6 (F and G). We then applied the same preprocessing procedure to each patient’s data separately to obtain log-transformed expression levels, which were used to generate Fig. 6H. Next, for each patient, we performed differential expression analyses between high and low CMV viral loads using three different statistical tests—Welch’s *t* test, Fisher’s exact test, and Mann-Whitney *U* test—and used the Benjamini-Hochberg procedure (*73*) to control the FDR at 5% for each test separately. We included all genes that are differentially expressed under any one of the three tests in table S9. All bulk and scRNA high-throughput sequencing data are available at the Gene Expression Omnibus (GSE132048).

### Statistical analysis

Graphs show a representative experiment of *n* ≥ 4 assays, with *n* ≥ 4 biological replicates. All statistical analyses were performed using GraphPad Prism version 7.04 (GraphPad Software Inc., La Jolla, CA). Depicted are means with SEM of the replicates unless otherwise stated. Differences were evaluated by the two-tailed Student’s *t* test when comparing two groups (unpaired *t* test when the two samples have unequal sample sizes) and two-way ANOVA with repeated measures with Bonferroni’s post-test when comparing more than two groups. Statistical significance was considered *P* < 0.05; *P* values and adjusted FDR *P* values are denoted as *P < 0.05, **P < 0.01, ***P < 0.001, and ****P < 0.0001. Nonsignificance was indicated by the letters ns.

## Supplementary Material

aax9856_Table_S6.xlsx

aax9856_Table_S4.xls

aax9856_Table_S9.xlsx

aax9856_Table_S7.xlsx

aax9856_Table_S3.xlsx

aax9856_SM.pdf

aax9856_Table_S5.xlsx

## SUPPLEMENTARY MATERIALS

Supplementary material for this article is available at http://advances.sciencemag.org/cgi/content/full/6/17/eaax9856/DC1

## Appendix

View/request a protocol for this paper from *Bio-protocol*.
