# Supplementary material for: Linking indirect effects of cytomegalovirus in transplantation to modulation of monocyte innate immune function
Source: Sci Adv. 2020 Apr 22;6(17):eaax9856. doi: 10.1126/sciadv.aax9856 (PMC7176434; doi:10.1126/sciadv.aax9856)
Supplement: aax9856_SM.pdf [file aax9856_SM.pdf]

Science Advances
AAAS

advances.sciencemag.org/cgi/content/full/6/17/eaax9856/DC1

Supplementary Materials for

Linking indirect effects of cytomegalovirus in transplantation to modulation of monocyte innate immune function

Pritha Sen, Adrian R. Wilkie, Fei Ji, Yiming Yang, Ian J. Taylor, Miguel Velazquez-Palafox, Emilia A. H. Vanni, Jean M. Pesola, Rosio Fernandez, Han Chen, Liza M. Morsett, Erik R. Abels, Mary Piper, Rebekah J. Lane, Suzanne E. Hickman, Terry K. Means, Eric S. Rosenberg, Ruslan I. Sadreyev, Bo Li, Donald M. Coen, Jay A. Fishman, Joseph El Khoury*

*Corresponding author. Email: jelkhoury@mgh.harvard.edu

Published 22 April 2020, *Sci. Adv.* **6**, eaax9856 (2020)
DOI: 10.1126/sciadv.aax9856

The PDF file includes:

Figs. S1 to S6
Tables S1 and S2
Legends for tables S3 to S7 and S9
Table S8

Other Supplementary Material for this manuscript includes the following:

(available at advances.sciencemag.org/cgi/content/full/6/17/eaax9856/DC1)

Tables S3 to S7 and S9

Supplementary Materials

Supplemental Table 1. Enriched pathways (MSigDB Hallmarks) in CMV-infected monocytes.

Supplemental Table 2. Enriched pathways (InnateDB) in CMV-infected monocytes.

Supplemental Table 3. Subcategorization of 154 unique GSEA pathways with significant normalized enrichment scores (NES) dysregulated in CMV-infected monocytes by immunological and/or biological function into "Summary" chord terms.

Supplemental Table 4. Differential gene expression table (log fold-change, FDR, CPM) for each detected transcript within CMV-infected $CD14^{+}/16^{-}$ and $CD14^{+}/16^{+}$ monocytes compared to mock-infected $CD14^{+}/16^{-}$ and $CD14^{+}/16^{+}$.

Supplemental Table 5. Differential gene expression table (log fold-change, FDR, CPM) in $CD14^{+}/16^{-}$ monocytes infected with live and UV-inactivated CMV. Comparisons between non-UV inactivated CMV (Live) and CMV subject to 10 (UV^{TC10}) and 60 (UV^{TC60}) minutes of direct UV light as compared to mock-infected monocytes are included.

Supplemental Table 6. Copies of GFP mRNA identified by bulk RNA-Seq in mock-, CMV- and UV-inactivated CMV-infected monocytes.

Supplemental Table 7. Differential gene expression table (log fold-change, FDR, CPM) of GFP and key host genes in mock, 10' and 60' UV-inactivated and live CMV-infected $CD14^{+}/16^{-}$ monocytes.

Supplemental Table 8. Percent fold change of key host genes in $CD14^{+}$ monocytes isolated from heart transplant recipients during detection of high versus low/undetectable CMV viral load. For comparison, directionality of expression (+ increased expression, - decreased expression) from our *in vitro* model is concurrently shown.

Supplemental Table 9. Differential gene expression table (log fold-change, percent fold-change, WAD score, Fisher q value) in $CD14^{+}$ monocytes isolated at high and low CMV viremia from cardiac transplant recipients CT00A, CT00B, CT00C, and CT00D.

Supplemental Fig 1. CMV-infected $CD14^{-}/16^{-}/HLA\text{-}DR^{+}/GFP^{+}$ circulating dendritic cells were identified by flow cytometry in this *in vitro* model of CMV infection.

Supplemental Fig 2. RNA-Seq was performed on $calcein^{+}/CD3/19/20/56^{-}/CD14^{+}/16^{-}$ and $CD14^{+}/16^{-}$ cells from mock-infected PBMC and $CD14^{+}/16^{-}/GFP^{+}$ and $CD14^{+}/16^{+}/GFP^{+}$ cells from CMV-infected PBMC from ten unique immunocompetent donors. Mock and CMV-GFP^{+} infected monocytes cluster in distinct populations as shown by **A,** correlation heatmap and **B,** PCA plot. **C,** One specimen from the mock-infected $CD14^{+}/16^{-}$ monocyte group (31F) was excluded based on having a lower number of CPM reads compared to all other mock specimens and two specimens from the CMV-infected $CD14^{+}/16^{-}$ monocyte group (27F, 33F, open black circles) were also excluded from the final analysis as they were relative outliers on PCA plot and **D,** found to have 10-fold greater number of copies of viral RNA and GFP compared to other biological replicates.

Supplemental Fig 3. Of the 38 CMV-associated viral transcripts detected in GFP^+ TB40/E-5 infected monocytes, 23 (60%) belonged to immediate-early and delayed-early families of transcripts and 12 (30%) belonged to the late family of transcripts. Immediate early (IE), delayed early (DE), leaky late (LL), true late (TL), latent (LAT).

Supplemental Fig 4. Chord diagram illustrating that the top 200 differentially expressed transcripts in CMV-infected $CD14^+/16^-$ monocytes spanned multiple different cellular compartments in the following distribution: extracellular (60), membrane-associated (67), cytosol (61), nuclear/transcription factor (43), Golgi/ER-phagolysosome (16), mitochondria (7), cytoskeleton (3) and lipoprotein (3).

Supplemental Fig 5. Percent of monocytes capable of phagocytosing pHrodo-red labeled *C. albicans* and *C. neoformans* as measured by conventional flow cytometry. There was a significant decrease in the overall percentage of CMV-infected monocytes phagocytosing pHrodo-labeled *C. neoformans* (Cn) (p value 0.01, paired t-test) but no significant difference in the overall percentage of monocytes actively phagocytosing pHrodo-red labeled *C. albicans* (Ca) (p value 0.54, paired t-test). p values are denoted ** = $p<0.01$]. Nonsignificance was indicated by the letters ns.

Supplemental Fig 6. Direct exposure to UV light successfully inactivates TB40/E-5. Quantitative RT-PCR and IHC for GFP and IE1, Western blot for GFP and UL84, and flow cytometry for GFP were performed on HFF infected at MOI of 1 with TB40/E-5 subjected to direct UV light at a distance of 6 inches from UV light source for 60 minutes (UV^{TC}) and one pulse of 360 mJ/cm^2 via Stratalinker (UV^{SL}). Compared to UV^{SL}, UV^{TC}-inactivated TB40/E-5 had a) decreased RNA expression levels of GFP and IE1 at 24 hours, b) abrogation of GFP and IE1 protein expression detected by immunohistochemistry at 24 hours and c) absence of GFP and UL84 protein expression detected by Western blot at 24 and 72 hours. d) Flow cytometry of HFF and PBMC infected with UV^{TC}-inactivated TB40/E-5 demonstrates absence of GFP^+ HFF; the percentage of GFP^+ monocytes is equivalent in UV^{TC}-irradiated and Live-TB40/E-5-infected PBMC, but the GFP MFI in monocytes infected with UV^{TC}-inactivated TB40/E-5 was diminished compared to monocytes infected with live virus.

Supplemental Fig. 1

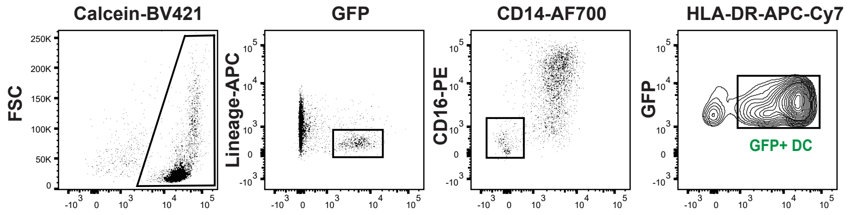

Supplemental Fig. 2

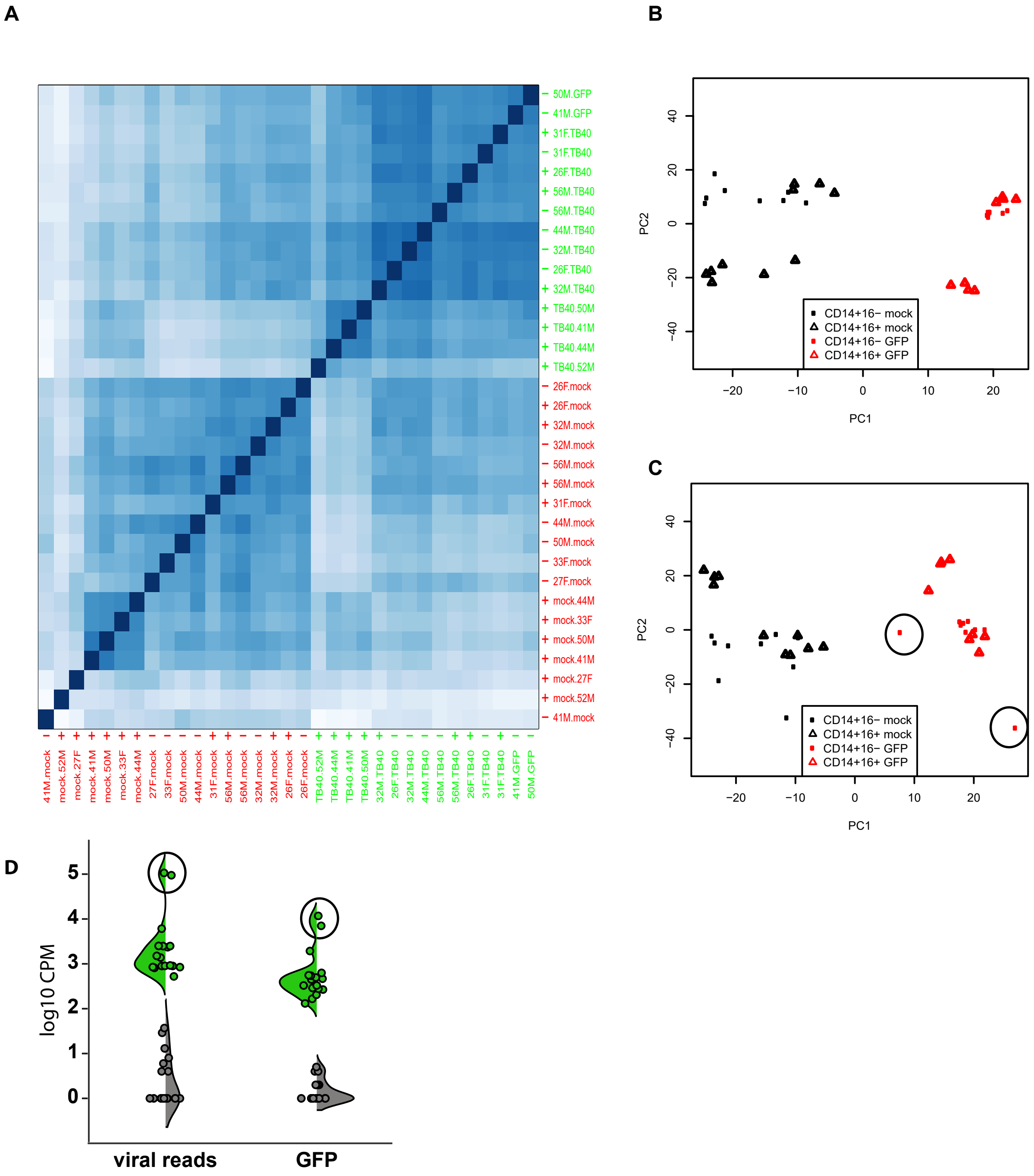

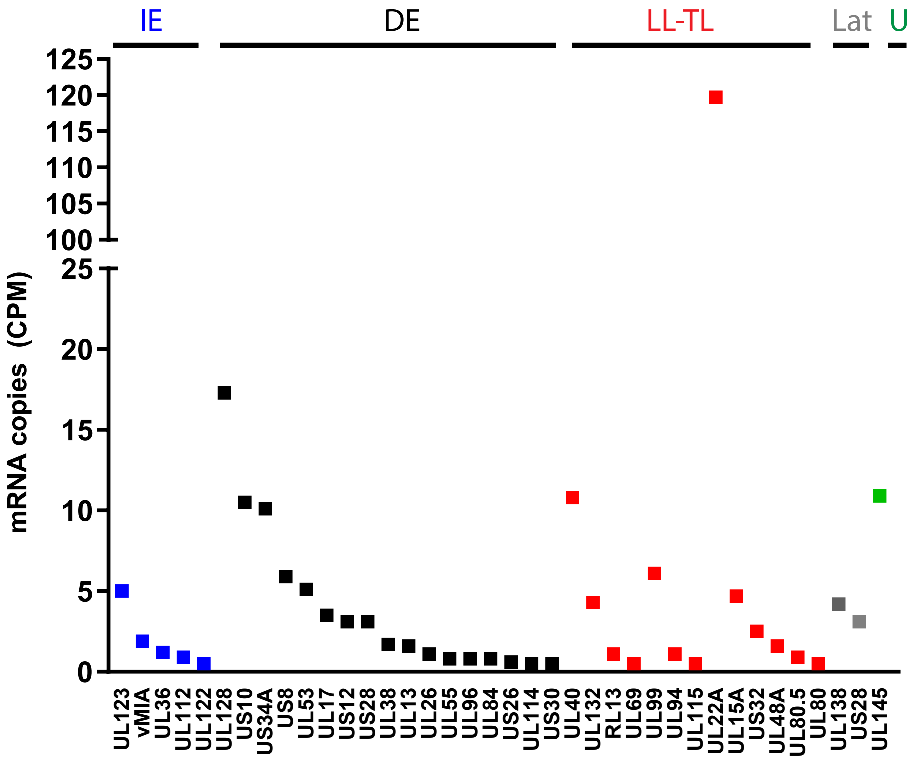
IE
DE
LL-TL
Lat
U
mRNA copies (CPM)
125
120
115
110
105
100
25
20
15
10
5
0
UL123
vMIA
UL36
UL112
UL122
UL128
US10
US34A
US8
UL53
UL17
US12
US28
UL38
UL13
UL26
UL55
UL96
UL84
US26
UL114
US30
UL40
UL132
RL13
UL69
UL99
UL94
UL115
UL22A
UL15A
US32
UL48A
UL80.5
UL80
UL138
US28
UL145

Supplemental Fig.4

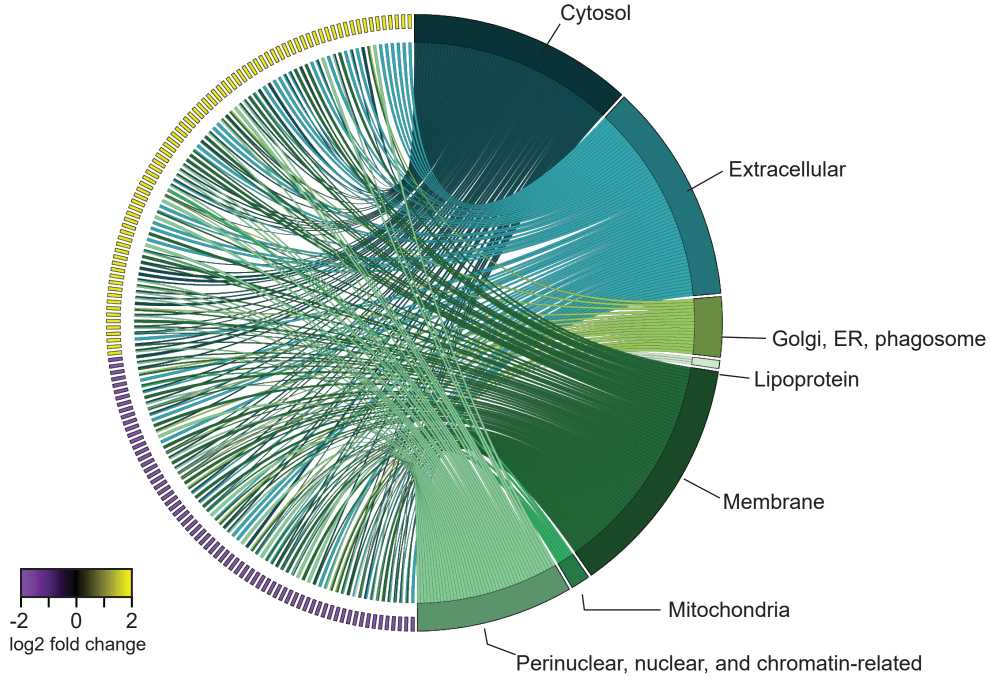

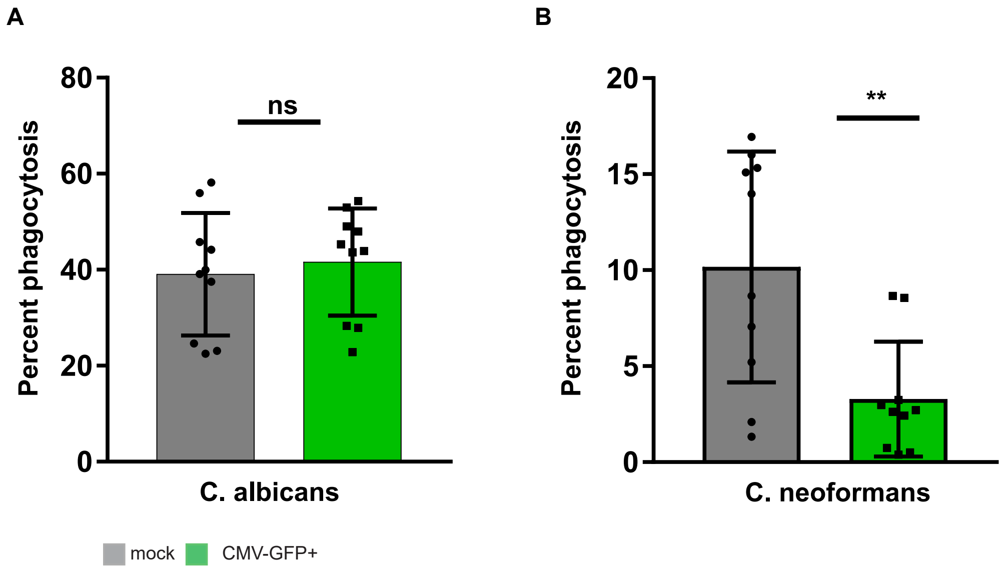
A
B
Percent phagocytosis
80
60
40
20
0
ns
C. albicans
20
15
10
5
0
**
C. neoformans
mock
CMV-GFP+

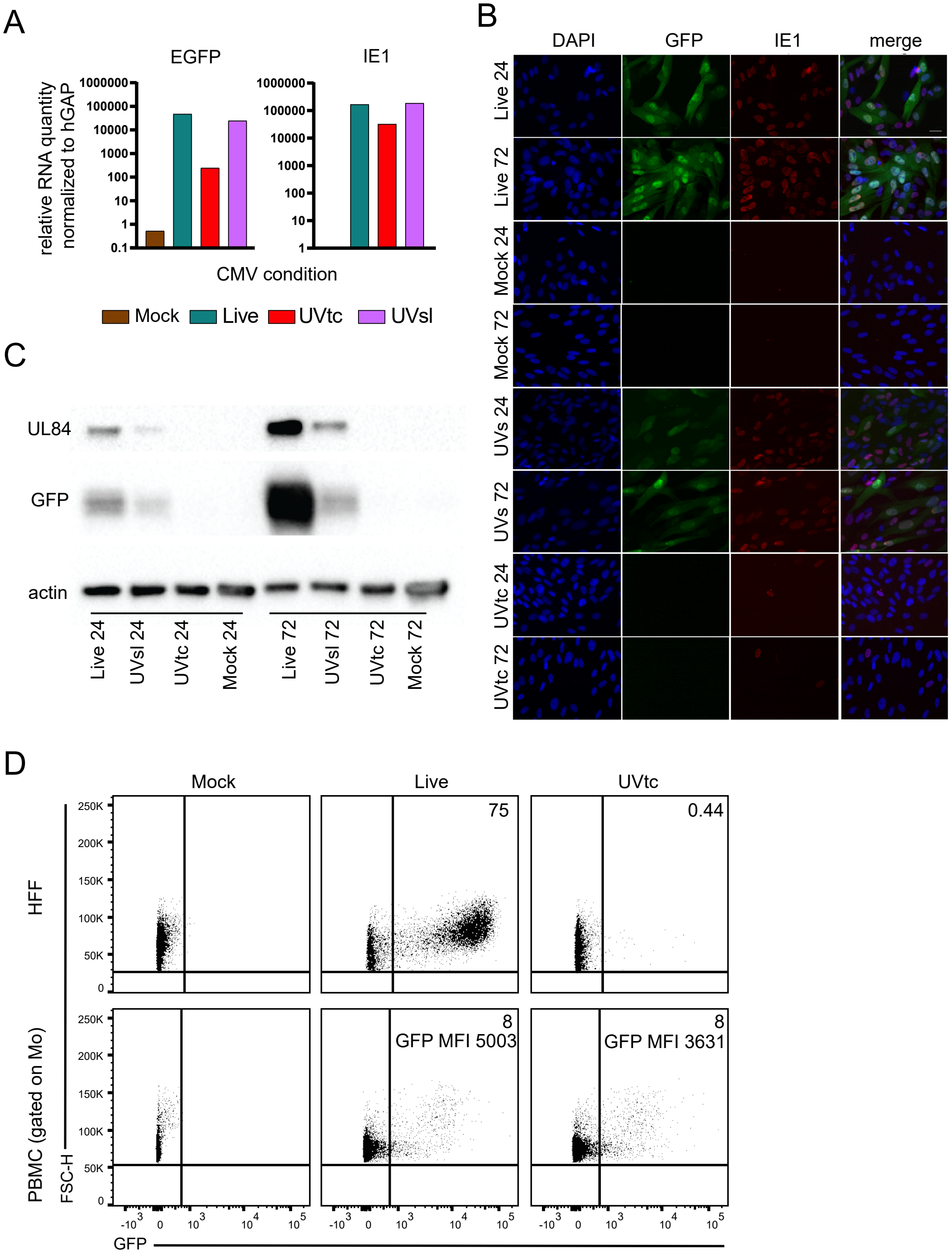
A
EGFP
IE1
relative RNA quantity normalized to hGAP
CMV condition
Mock
Live
UVtc
UVsl
B
DAPI
GFP
IE1
merge
Live 24
Live 72
Mock 24
Mock 72
UVs 24
UVs 72
UVtc 24
UVtc 72
C
UL84
GFP
actin
Live 24
UVsl 24
UVtc 24
Mock 24
Live 72
UVsl 72
UVtc 72
Mock 72
D
Mock
Live
UVtc
HFF
PBMC (gated on Mo)
FSC-H
GFP
75
0.44
8
GFP MFI 5003
8
GFP MFI 3631

Supplemental Table 1

Pathway Name	NES	FDR q-val
HALLMARK_ADIPOGENESIS	-2.012	0.00E+00
HALLMARK_ALLOGRAFT_REJECTION	1.486	2.20E-02
HALLMARK_ANDROGEN_RESPONSE	-1.446	4.33E-02
HALLMARK_ANGIOGENESIS	-2.037	0.00E+00
HALLMARK_APICAL_SURFACE	1.579	1.16E-02
HALLMARK_APOPTOSIS	1.709	6.13E-03
HALLMARK_CHOLESTEROL_HOMEOSTASIS	-1.476	3.84E-02
HALLMARK_COAGULATION	-1.604	1.24E-02
HALLMARK_COMPLEMENT	1.726	5.91E-03
HALLMARK_DNA_REPAIR	-1.276	1.36E-01
HALLMARK_FATTY_ACID_METABOLISM	-1.706	3.86E-03
HALLMARK_GLYCOLYSIS	-1.351	8.54E-02
HALLMARK_IL2_STAT5_SIGNALING	1.616	1.11E-02
HALLMARK_IL6_JAK_STAT3_SIGNALING	1.345	5.60E-02
HALLMARK_INFLAMMATORY_RESPONSE	2.620	0.00E+00
HALLMARK_INTERFERON_ALPHA_RESPONSE	3.632	0.00E+00
HALLMARK_INTERFERON_GAMMA_RESPONSE	3.846	0.00E+00
HALLMARK_KRAS_SIGNALING_DN	1.442	2.70E-02
HALLMARK_MTORC1_SIGNALING	-1.777	1.69E-03
HALLMARK_MYC_TARGETS_V1	-2.329	0.00E+00
HALLMARK_MYC_TARGETS_V2	-1.504	3.34E-02
HALLMARK_MYOGENESIS	-1.458	4.17E-02
HALLMARK_OXIDATIVE_PHOSPHORYLATION	-2.641	0.00E+00
HALLMARK_PEROXISOME	-2.128	0.00E+00
HALLMARK_PI3K_AKT_MTOR_SIGNALING	-1.501	3.12E-02
HALLMARK_TGF_BETA_SIGNALING	-1.705	3.38E-03
HALLMARK_TNFA_SIGNALING_VIA_NFKB	2.300	0.00E+00
HALLMARK_XENOBIOTIC_METABOLISM	-1.341	8.25E-02

Supplemental Table 2

Up	InnateDB Pathway Name	Pathway	Source	p-value	p-adj
UP	FasL/ CD95L signaling	13409	REACTOME	4.8E-02	2.9E-01
UP	Free fatty acid receptors	13242	REACTOME	4.8E-02	2.9E-01
UP	Apoptotic cleavage of cell adhesion proteins	13416	REACTOME	3.4E-02	2.5E-01
UP	Direct p53 effectors	15209	PID NCI	3.1E-02	2.4E-01
UP	Il-2 receptor beta chain in t cell activation	4011	PID BIOCARTA	2.8E-02	2.3E-01
UP	TNFR1 signaling pathway	16212	INOH	2.5E-02	2.1E-01
UP	NOD-like receptor signaling pathway	8112	KEGG	2.5E-02	2.2E-01
UP	Hiv-1 nef: negative effector of fas and tnf	4101	PID BIOCARTA	1.4E-02	1.5E-01
UP	NOD1/2 Signaling Pathway	13056	REACTOME	1.3E-02	1.5E-01
UP	Caspase-8 activation by cleavage	13394	REACTOME	1.2E-02	1.3E-01
UP	Dimerization of procaspase-8	13393	REACTOME	1.2E-02	1.3E-01
UP	Regulation by c-FLIP	19810	REACTOME	1.2E-02	1.3E-01
UP	IL5-mediated signaling events	15880	PID NCI	9.4E-03	1.2E-01
UP	Il 3 signaling pathway	3967	PID BIOCARTA	9.4E-03	1.2E-01
UP	Internal ribosome entry pathway	3997	PID BIOCARTA	8.7E-03	1.2E-01
UP	TRAF6 mediated NF-kB activation	13015	REACTOME	7.2E-03	9.8E-02
UP	TNF signaling	13410	REACTOME	7.0E-03	9.8E-02
UP	TRAIL signaling	13411	REACTOME	7.0E-03	9.8E-02
UP	Activation of IRF3/IRF7 mediated by TBK1/IKK epsilon	13028	REACTOME	6.7E-03	9.7E-02
UP	Tnfr1 signaling pathway	4052	PID BIOCARTA	6.7E-03	9.7E-02
UP	The NLRP3 inflammasome	13060	REACTOME	6.7E-03	1.0E-01
UP	Caspase cascade in apoptosis	4166	PID BIOCARTA	5.7E-03	8.8E-02
UP	Viral RNP Complexes in the Host Cell Nucleus	13705	REACTOME	5.6E-03	9.0E-02
UP	Inflammasomes	13061	REACTOME	5.0E-03	8.3E-02
UP	GMCSF-mediated signaling events	15598	PID NCI	4.5E-03	7.7E-02
UP	TRIF-mediated programmed cell death	13029	REACTOME	4.5E-03	7.8E-02
UP	Toll Like Receptor 4 (TLR4) Cascade	13046	REACTOME	4.0E-03	7.2E-02
UP	Growth hormone signaling	16149	INOH	2.9E-03	5.3E-02
UP	TRAIL signaling pathway	15206	PID NCI	2.7E-03	5.0E-02
UP	TRAF3-dependent IRF activation pathway	13014	REACTOME	2.6E-03	5.0E-02
UP	Adaptive Immune System	18371	REACTOME	2.5E-03	4.9E-02
UP	IL2	15918	NETPATH	2.5E-03	4.9E-02
UP	HIV-1 Nef: Negative effector of Fas and TNF-alpha	15863	PID NCI	2.4E-03	4.9E-02
UP	Negative regulators of RIG-I/MDA5 signaling	13013	REACTOME	2.4E-03	4.9E-02
UP	IL27-mediated signaling events	15133	PID NCI	2.4E-03	4.9E-02
UP	MyD88-independent cascade	13030	REACTOME	2.1E-03	4.5E-02
UP	TRIF-mediated TLR3/TLR4 signaling	19507	REACTOME	2.1E-03	4.5E-02
UP	Toll Like Receptor 3 (TLR3) Cascade	13031	REACTOME	2.1E-03	4.5E-02
UP	Fas signaling pathway (cd95)	4088	PID BIOCARTA	2.0E-03	4.5E-02
UP	FAS (CD95) signaling pathway	15900	PID NCI	1.8E-03	4.3E-02
UP	Trafficking and processing of endosomal TLR	13047	REACTOME	1.8E-03	4.2E-02
UP	Caspase Cascade in Apoptosis	14995	PID NCI	1.6E-03	3.8E-02
UP	Oncostatin_M	16513	NETPATH	1.5E-03	3.7E-02
UP	Induction of apoptosis through dr3 and dr4/5 death receptors	4039	PID BIOCARTA	1.4E-03	3.6E-02
UP	Antiviral mechanism by IFN-stimulated genes	17226	REACTOME	1.4E-03	3.6E-02
UP	ISG15 antiviral mechanism	13075	REACTOME	1.4E-03	3.6E-02
UP	IL2-mediated signaling events	15044	PID NCI	1.3E-03	3.5E-02
UP	IL23-mediated signaling events	15427	PID NCI	1.3E-03	3.5E-02
UP	Activated TLR4 signalling	19190	REACTOME	1.3E-03	3.5E-02
UP	NF-kB activation through FADD/RIP-1 pathway mediated by caspase-8 and -10	13016	REACTOME	1.2E-03	3.4E-02
UP	Tnfr2 signaling pathway	4004	PID BIOCARTA	1.2E-03	3.4E-02
UP	STING mediated induction of host immune responses	13011	REACTOME	1.0E-03	3.2E-02
UP	EGFR1	15908	NETPATH	1.0E-03	3.2E-02
UP	Nucleotide-binding domain, (NLR) signaling pathways	18305	REACTOME	9.5E-04	3.1E-02
UP	Activation of C3 and C5	13049	REACTOME	9.0E-04	3.0E-02
UP	Cyclin A/B1 associated events during G2/M transition	12973	REACTOME	8.0E-04	2.7E-02
UP	Regulation of innate immune responses to cytosolic DNA	18110	REACTOME	7.1E-04	2.5E-02
UP	Toll-Like Receptors Cascades	17767	REACTOME	6.7E-04	2.4E-02
UP	Immunoregulatory interactions between a Lymphoid and a non-Lymphoid cell	13066	REACTOME	6.6E-04	2.5E-02
UP	RIP-mediated NFkB activation via ZBP1	13008	REACTOME	5.8E-04	2.2E-02
UP	Signaling by Interleukins	18744	REACTOME	4.7E-04	1.8E-02
UP	Regulation of autophagy	431	KEGG	4.4E-04	1.8E-02
UP	ZBP1(DAI) mediated induction of type I IFNs	13010	REACTOME	4.4E-04	1.9E-02
UP	Chemokine receptors bind chemokines	13243	REACTOME	4.3E-04	1.9E-02
UP	Factors involved in megakaryocyte development and platelet production	13101	REACTOME	3.3E-04	1.5E-02
UP	KitReceptor	15910	NETPATH	2.2E-04	1.1E-02
UP	Chemokine signaling pathway	4389	KEGG	1.7E-04	8.6E-03
UP	Cytosolic sensors of pathogen-associated DNA	19557	REACTOME	9.7E-05	5.0E-03
UP	Natural killer cell mediated cytotoxicity	578	KEGG	8.4E-05	4.5E-03
UP	Autoimmune thyroid disease	2799	KEGG	5.4E-05	3.1E-03
UP	Death Receptor Signalling	19890	REACTOME	2.7E-05	1.6E-03
UP	Extrinsic Pathway for Apoptosis	17266	REACTOME	2.7E-05	1.6E-03
UP	Growth hormone receptor signaling	13072	REACTOME	1.4E-05	9.4E-04
UP	JAK STAT pathway and regulation	16125	INOH	3.3E-07	2.3E-05
UP	Toll-like receptor signaling pathway	564	KEGG	9.6E-08	7.2E-06
UP	IFN alpha signaling	16239	INOH	3.4E-08	2.7E-06
UP	Interferon gamma signaling	13077	REACTOME	1.4E-08	1.2E-06
UP	Hepatitis C	10404	KEGG	1.4E-08	1.3E-06
UP	Regulation of IFNA signaling	13073	REACTOME	9.0E-09	8.9E-07
UP	Cytosolic DNA-sensing pathway	8117	KEGG	5.5E-09	6.0E-07
UP	TRAF6 mediated IRF7 activation	13012	REACTOME	1.1E-09	1.3E-07
UP	Innate Immune System	17476	REACTOME	3.6E-10	4.8E-08
UP	RIG-I/MDA5 mediated induction of IFN-alpha/beta pathways	13017	REACTOME	2.8E-11	4.1E-09
UP	Jak-STAT signaling pathway	568	KEGG	1.4E-11	2.4E-09
UP	Cytokine-cytokine receptor interaction	515	KEGG	7.2E-12	1.4E-09
UP	RIG-I-like receptor signaling pathway	5713	KEGG	1.6E-12	3.8E-10
UP	Immune System	18444	REACTOME	1.6E-20	4.8E-18
UP	Interferon Signaling	18059	REACTOME	5.0E-21	2.0E-18
UP	Interferon alpha/beta signaling	13074	REACTOME	1.2E-23	7.3E-21
UP	Cytokine Signaling in Immune system	17418	REACTOME	2.7E-24	3.2E-21

Down	InnateDB Pathway Name	Pathway	Source	p-value	p-adj
DOWN	Regulation of KIT signaling	13324	REACTOME	4.8E-02	2.4E-01
DOWN	Signaling by FGFR in disease	16656	REACTOME	3.4E-02	2.1E-01
DOWN	Downstream signaling of activated FGFR	16879	REACTOME	2.8E-02	1.9E-01
DOWN	VEGFR1 specific signals	15709	PID NCI	2.1E-02	2.1E-01
DOWN	Signaling by EGFR	13216	REACTOME	2.0E-02	2.1E-01
DOWN	Endogenous TLR signaling	15845	PID NCI	1.7E-02	1.8E-01
DOWN	Signaling by FGFR	13320	REACTOME	1.3E-02	1.7E-01
DOWN	DAP12 interactions	13004	REACTOME	1.2E-02	1.7E-01
DOWN	Biosynthesis of unsaturated fatty acids	488	KEGG	9.8E-03	1.6E-01
DOWN	Staphylococcus aureus infection	10357	KEGG	6.7E-03	1.5E-01
DOWN	Alpha4 beta1 integrin signaling events	15573	PID NCI	4.6E-03	1.3E-01
DOWN	Tryptophan metabolism	504	KEGG	3.7E-03	1.2E-01
DOWN	Retinoid metabolism and transport	13323	REACTOME	1.3E-03	5.9E-02
DOWN	Cross-presentation of particulate exogenous antigens (phagosomes)	12984	REACTOME	1.0E-03	4.9E-02
DOWN	Integrin cell surface interactions	13303	REACTOME	8.5E-04	4.3E-02
DOWN	Response to elevated platelet cytosolic Ca2+	13131	REACTOME	8.5E-04	4.3E-02
DOWN	Phagosome	10394	KEGG	8.2E-04	4.6E-02
DOWN	Beta3 integrin cell surface interactions	15634	PID NCI	5.9E-04	3.4E-02
DOWN	Urokinase-type plasminogen activator (uPA) and uPAR-mediated signaling	15219	PID NCI	5.9E-04	3.4E-02
DOWN	Extracellular matrix organization	17095	REACTOME	5.4E-04	3.5E-02
DOWN	Beta5 beta6 beta7 and beta8 integrin cell surface interactions	15811	PID NCI	4.9E-04	3.4E-02
DOWN	Eicosanoid metabolism	3989	PID BIOCARTA	4.7E-04	3.4E-02
DOWN	Platelet degranulation	13130	REACTOME	4.5E-04	3.5E-02
DOWN	Hemostasis	19856	REACTOME	3.9E-04	3.2E-02
DOWN	FGF signaling pathway	15346	PID NCI	3.5E-04	3.2E-02
DOWN	Syndecan interactions	17559	REACTOME	2.3E-04	2.2E-02
DOWN	Alpha9 beta1 integrin signaling events	15241	PID NCI	1.2E-04	1.4E-02
DOWN	Beta1 integrin cell surface interactions	14944	PID NCI	1.0E-04	1.3E-02
DOWN	Valine Leucine Isoleucine degradation	16210	INOH	8.2E-05	1.2E-02
DOWN	Cell surface interactions at the vascular wall	13112	REACTOME	5.2E-05	8.7E-03
DOWN	Metabolism of lipids and lipoproteins	16920	REACTOME	2.9E-05	5.7E-03
DOWN	PPAR signaling pathway	566	KEGG	6.4E-06	1.5E-03
DOWN	Hematopoietic cell lineage	415	KEGG	2.8E-06	8.1E-04
DOWN	Integrins in angiogenesis	15117	PID NCI	2.6E-06	1.0E-03
DOWN	Integrin family cell surface interactions	15169	PID NCI	2.6E-06	1.5E-03
DOWN	Metabolism	19429	REACTOME	5.3E-08	6.2E-05

	Subject	CMV-00A	CMV-00B	CMV-00C	CMV-00D	*in vitro*
	**VL (IU/ml)**	58,900 → 562	147 → 1500	366,000 → 238	55,000 → 137	NA
	**days lapsed**	47	49	28	35	1
**Viral PRR**	**DHX58**		2.07	1.66	3.89	+
	**DDX58**	1.83	4.95		5.36	
	**IFIH1**	1.72	3.05	1.87	8.26	
	**ZBP1**	1.78	1.57	2.09	4.07	
	**NOD1**			0.64	3.67	
	**NOD2**				1.74	
**Fungal PRR**	**CD14**		0.86	1.56	1.18	-
	**CD36**	0.85		1.19	1.21	
	**ITGB2**		0.98	1.011000037	1.07	
	**ITGAM**	0.76		1.133000016		
	**STAB1**	0.61			1.21	
**Inflammasome**	**AIM2**	3.29	5.93	1.508000016	11.82	+
	**IFI16**	1.52	2.49	0.750999987	2.65	
	**MEFV**	1.09	1.74	0.833000004	1.70	
	**CASP1**		1.30	0.867999971	1.59	
	**CASP4**		1.48	0.818000019	1.53	
**Rejection**	**CXCL11**				12.50	+
	**CCL8**					
	**IDO1**	7.25			11.92	
	**WARS**	1.24	1.36	1.02	1.05	
	**TAP1**	1.57	1.73	1.67	3.01	
**ISG**	**MX1**	1.19	2.92	2.77	5.47	+
	**MX2**	1.23	2.06	1.44	3.44	
	**EIF2AK2**	1.14	1.92	2.49	2.57	
	**OAS1**	1.47	2.44	1.21	4.04	
	**IRF7**	1.21	1.73	2.15	3.09	
